# A Reproducible Benchmark-Validity Audit and Calibration Study for Cross-Home Fault Diagnosis in Smart-Home Sensor Systems

**DOI:** 10.3390/s26165025

**Published:** 2026-08-07

**Authors:** Norkobil Saydirasulovich Saydirasulov, Abror Shavkatovich Buriboev, Shuxrat Isroilov, Ryumduck Oh, Shavkat Buribayev, Abbos Abduvaytov, Jamshid Umirov, Jasur Ismailovich Badalov, Aziza Axmedova, Cheolwon Lee, Heung Seok Jeon

**Affiliations:** 1 Department of Computer Engineering, Gachon University, Seongnam-si 13120, Republic of Korea; saydirasulov@gachon.ac.kr; 2 Department of Engineering and Communications, ZARMED University, Samarkand Campus, Khodja Akhror Vali Street 23, Samarkand 140100, Uzbekistan; 3 Department of Artificial Intelligence, Samarkand State University, Samarkand 140104, Uzbekistan; abror1989@gachon.ac.kr (A.S.B.); i.shuha84@gmail.com (S.I.); 4 Department of Computer Engineering, Korea National University of Transportation, Chungju-si 27469, Republic of Korea; rdoh@ut.ac.kr; 5 Department of Construction Engineering, Samarkand State Technical University, Samarkand 140100, Uzbekistan; abbosshav@gmail.com; 6 Department of Information Technology, Samarkand Institute of Economics and Service, Samarkand 140100, Uzbekistan; abbosabduvaytov069@gmail.com (A.A.); umirov07@sies.uz (J.U.); 7 Department of Radio and Mobile Communications, Tashkent University of Information Technologies, Tashkent 100084, Uzbekistan; badalov@tuit.uz; 8 Department of Exact Sciences, Kimyo International University in Tashkent, Tashkent 100121, Uzbekistan; aziza_axmedova@kiut.uz; 9 Department of Computer Engineering, Konkuk University, Chungju-si 27478, Republic of Korea; cheolwonlee@kku.ac.kr

**Keywords:** artificial intelligence, intelligent sensors, Internet of Things (IoT), data fusion, smart home, anomaly diagnosis, counterfactual explanation, calibration, causal inference, sensor fault detection

## Abstract

Diagnosing faults across different smart homes is hard: sensor names, layouts, and daily routines differ from home to home, so a model trained in one home rarely works in another. We study an ontology-guided framework for cross-home fault diagnosis, but our main contribution is a benchmark-validity audit—a systematic check of whether the datasets used to evaluate such systems actually measure fault detection. Using the public Center for Advanced Studies in Adaptive Systems (CASAS) smart-home datasets (homes hh101–hh110) and real household power data (HomeC, UMass Smart*), we show that much of the high cross-home accuracy reported on these benchmarks is an artifact of features that re-encode the labelling rules rather than evidence of transfer: when those features are removed, the macro-averaged F1 score (macro-F1) collapses toward the level obtained with randomly permuted labels. We therefore treat these datasets as semantic-transfer and benchmark-validity studies, not fault-detection results. The framework’s distinguishing component is a counterfactual calibration layer that returns a probability for its recommended intervention; on a controlled structural causal model with known interventions, it achieves a Brier skill score of 0.369 for intervention-success probabilities. Separately, on the simulation-derived LBNL Fan Coil Unit benchmark, a conventional gradient-boosted multiclass fault classifier achieves accuracy comparable to a random forest but about six times lower expected calibration error (0.026 vs. 0.159) under a scenario-matched split. This calibration advantage does not generalize to held-out simulation scenarios, where the calibration error rises to 0.372; we report this negative result as a limitation. We are explicit about scope: the ontology reasoner and the real-stream causal graph are only partially implemented, and the counterfactual recommendations are validated only under controlled or simulated conditions, not in deployed homes. The results are intended for researchers who build or benchmark sensor-based fault-diagnosis models, for dataset curators, and for practitioners who need calibrated rather than merely accurate outputs. All code, the proxy-label rules, and the leakage audit are released.

## 1. Introduction

A modern instrumented home can hold anywhere from a few dozen to more than a hundred sensors—motion detectors, contact sensors on doors and cabinets, thermostats, and appliance monitors; the CASAS research homes used here, for example, expose on the order of tens of sensor channels per home. Most of these systems still run on simple logic. They follow schedules, answer voice commands, and trigger a rule when a reading crosses a threshold, but they do not interpret what they record. Take a window left open while the air conditioner works against it, an occupancy sensor that disagrees with the door state during a security-mode change, or a motion sensor that decays into a fixed-period signal. At best, the home raises an alert; often it does nothing [1,2]. What residents really need is harder to provide: an explanation of why the event happened, and a suggested action to fix it. That need is most pressing in the settings where smart homes are supposed to matter most, including safety monitoring, aging-in-place, and energy management [3,4].

Most existing work is aimed at a different question. Activity recognition asks what the resident is doing (sleeping, cooking, leaving the house) and a long line of transfer-learning, zero-shot, and layout-agnostic methods has refined that task considerably [5,6,7,8,9,10]. These approaches classify events; they do not diagnose them. The problem we care about is the inverse: an unexpected joint configuration of sensors and devices that signals a fault, which falls outside the object of study of activity recognition [5,6,11]. The knowledge-representation side has a complementary gap. Brick [12] captures building topology and SAREF [13] captures device semantics; both are rigorous and widely used, but neither was designed to express abnormality classes, diagnostic causal links, or the semantic portability that cross-home reasoning requires. They describe how a home is wired, not how it can go wrong.

The cross-home setting is the hardest. Homes differ in layout, sensor placement, device inventory, and resident behavior, so a model fitted to one home rarely transfers without retraining on local data [6,8,9,10]. Three separate lines of research each address a fragment of the larger problem: semantic representation, causal diagnosis, and intervention-oriented explanation. Individual pairs have been combined—causal root-cause analysis over device-interaction graphs in smart homes [14,15] and ontology- or rule-driven diagnosis of building systems [16,17]—and we build on that work rather than claim priority over it. What we do not find is a single framework that integrates all three for cross-home diagnosis with a calibrated intervention layer; we treat that as a scoping observation that motivates the design, not as a strong novelty claim, and Section 3.2 makes the specific gaps explicit.

This paper specifies and partially validates such a framework, whose three-layer architecture is presented in Section 3. Raw observations from each home are mapped onto a shared semantic vocabulary through a lightweight, home-specific reconfiguration. The normalized features then feed a discrete causal graph whose nodes span rooms, devices, user states, goal states, and abnormality classes, and whose edges are set by ontology constraints; diagnosis runs over this graph by belief propagation. A counterfactual stage follows: for any predicted abnormality, the system proposes an intervention together with a calibrated probability that the intervention will resolve it, validated against held-out outcomes. Because the semantic abstraction layer strips home-specific identifiers early, the downstream inference never sees raw device IDs.

The study is organized around three research questions. **RQ1:** when a cross-home fault-diagnosis benchmark is annotated by proxy rules rather than by experts, how much of the reported cross-home performance reflects semantic transfer and how much reflects recovery of the annotation rule itself? **RQ2:** does that effect depend on the model family, or is it a property of the feature representation? **RQ3:** can a structured counterfactual layer deliver intervention-success probabilities that are better calibrated than those of a strong flat classifier, and under which evidential conditions do those probabilities hold? Section 5.1, Section 5.2 and Section 5.3 address RQ1, RQ2 and RQ3 in turn.

We separate the contributions into three kinds, so that results demonstrated here are not read as claims about the parts that remain proposed.

1. **Demonstrated findings.** (i) On CASAS hh101–hh110, the reported cross-home F1 is largely a proxy-label leakage artifact: removing the features that re-encode the disclosed labelling rules collapses macro-F1 toward the permuted-label floor, and under a shared feature set, the model family is not the deciding factor. (ii) The same proxy-label circularity recurs on real appliance data (HomeC, UMass Smart*: a rule-exposed macro-F1 of 0.963 collapsing to a near-floor 0.445). (iii) A factor-graph counterfactual layer returns calibrated intervention-success probabilities on a controlled structural causal model (Brier skill score 0.369), and a gradient-boosted classifier is well-calibrated on the simulation-derived LBNL FDD benchmark under the scenario-matched split (ECE 0.026), though this calibration does not survive a held-out-scenario protocol (Section 5.5).
2. **A methodological insight.** We package the above into a reusable benchmark-validity audit protocol—rule-blind feature ablation, leave-one-rule-out, permuted-label controls, and a shared-feature comparison across model families—that lets others test whether a smart-home benchmark measures fault detection or merely label-rule recovery before reporting accuracy on it.
3. **A proposed architecture, validated in part.** We propose an extension to Brick and SAREF with abnormality-pattern classes and four diagnostic causal-relation types, and a composable three-layer design (Algorithm 1). *Implemented and evaluated:* a machine-checkable OWL/SHACL ontology with reasoner-consistency and competency-question results (Appendix A), the calibration layer, and an executable ontology prototype under controlled fault injection. *Specified but not yet implemented end-to-end:* the ontology-derived probabilistic causal graph on real streams, its fitted conditional probability tables and factor potentials, loopy belief propagation over real episodes, and the complete four-component tuple—including a calibrated probability—on real homes. Algorithm 1 describes the intended end-to-end pipeline; it is not run as a single system in the present experiments.

This paper is a methodological study of semantic transfer, benchmark validity, and calibrated counterfactual diagnosis, not a claim of state-of-the-art fault-detection accuracy. The anomalies in the Center for Advanced Studies in Adaptive Systems (CASAS) homes hh101–hh110—a public third-party smart-home benchmark released by Washington State University [2], not data we collected—are defined by disclosed proxy rules (Section 4.3) rather than expert annotation, because expert annotations do not exist for these homes. CASAS is nonetheless the appropriate benchmark for this study precisely because it is the most widely used public multi-home corpus for cross-home evaluation: an honest audit of what such a benchmark can and cannot measure is more useful to the community than an unauditable result on a private dataset. Our benchmark-validity audit (Section 5.1) shows that the high cross-home F1 obtainable on this benchmark is largely an artifact of features that re-encode those rules. We therefore distinguish four label regimes throughout: (i) proxy-rule labels (CASAS), (ii) physics-derived heuristic labels on real measurements (HomeC), (iii) simulation-derived ground-truth labels (the Lawrence Berkeley National Laboratory Fault Detection and Diagnostics (LBNL FDD) datasets, generated with the HVACSIM+ building-systems simulator), and (iv) synthetic interventions with known effects (the structural causal model used for calibration). No result rests on expert-verified real-world faults, and the intervention-success calibration claim is supported only under regime (iv), while regime (iii) supports only the multiclass fault-probability calibration result. One further scope point belongs up front: because AbnormalDoorEvent occurs in only three of the 45,969 CASAS episodes, the CASAS audit is effectively over *two* proxy classes, SensorDegradation and OccupancyModeConflict; we state this here so it frames every CASAS number that follows. We state this scope at the outset so the framework’s contribution can be judged independently of benchmark limitations.
**Algorithm 1:** End-to-end cross-home diagnosis and intervention**Input:** per-home raw stream $X_{h}$; causal home ontology O; graph template *G*; thresholds $r_{\mathrm{rec}}=0.70$, $r_{\mathrm{auto}}=0.85$.**Output:** structured diagnosis $y_{j}=\left(a_{j},c_{j},q_{j},r_{j}\right)$ for each episode *j*.*Semantic normalization.* Map raw identifiers to ontology types, $S\leftarrow \varphi_{h}\left(X_{h}\right)$, and discretize each variable (Section 3.3).*Windowing.* Segment *S* into episodes $W_{j}$ (k=15, stride s=15) and form the feature vector $f_{j}$.*Graph instantiation.* Read off ontology-typed nodes and {causes, enables, prevents, indicates} edges to instantiate the DAG G=(V,E) for $W_{j}$.*Diagnosis.* Run damped loopy belief propagation to obtain the abnormality label $a_{j}$ and the most-probable cause $c^{*}=\arg\max_{c\in C}P\left(c|e,G\right)$.*Counterfactual ranking.* For each ontology-admissible intervention $z^{\prime }$, evaluate $\Delta P(Y=\mathrm{normal}|\mathrm{do}\left(z=z^{\prime }\right),e)$ and select the best as $q_{j}$.*Calibrated decision.* Return the Brier-calibrated success probability $r_{j}$; issue $q_{j}$ automatically if $r_{j}\geq r_{\mathrm{auto}}$, recommend it if $r_{j}\geq r_{\mathrm{rec}}$, otherwise abstain.

The remainder of the paper is organized as follows. Section 2 surveys activity recognition, smart-home ontologies, and causal diagnosis. Section 3 presents the framework. Section 4 describes the evaluation protocol and the synthetic-proxy disclosure rules. Section 5 reports the CASAS benchmark-validity audit, the shared-feature baselines, the SCM and LBNL FDD results. Section 6 discusses what works and what does not, and Section 7 concludes.

## 2. Related Work

### 2.1. Cross-Home Activity Transfer

Early smart-home research made one point clear: practical intelligence depends as much on the sensing infrastructure, the middleware, and the adaptive interaction layer as it does on any single inference algorithm [1,2]. Activity recognition has been the dominant downstream task: foundational human-activity-recognition (HAR) work established the multi-home datasets the field still relies on [18] and the setting-generalized activity models that followed [19], while transfer-learning, zero-shot, and layout-agnostic methods established a second lesson—semantic abstractions transfer between homes in ways that raw sensor identifiers do not [6,7,8,9,10]. *Open challenge:* this line transfers activity *recognition*, not fault *diagnosis*, and, as our audit shows, evaluations built on proxy labels can reward re-encoding the labelling rule rather than genuine transfer.

### 2.2. Smart-Home Ontologies

On the knowledge-representation side, E-care@home [20] and POLARIS [21] integrate heterogeneous sensor data through domain-specific ontologies, and Brick [12], SAREF [13], DogOnt [22], and the SSN/SOSA family [17,23,24] supply shared schemas for buildings and IoT devices. *Open challenge:* none of these models treats abnormality patterns or diagnosis-oriented causal relations as first-class objects; like their predecessors, they describe how a home is wired, not how it can fail.

### 2.3. Causal Fault Diagnosis in Smart Homes

A body of work sits closer to diagnosis. SmartFABER [16] argued that structural knowledge constraints become necessary once anomaly instances are sparse, which directly motivates our ontology constraints; change-point surveys [25] explained why behavioural transitions are hard to catch; ambient-assisted-living studies [26] moved the objective from recognition toward diagnosis; anomaly-detection work confirmed that temporal event intervals carry discriminative information [27]; and safety-monitoring studies [3,4] reinforced the case for cause identification and intervention over mere alerts. The closest contemporary work approaches attribution through causality: Ge et al. [14] propose a causality-driven framework for anomaly attribution in closed-source platforms; a device-interaction-graph approach [15] reports 99.07% accuracy on a synthetic-injection benchmark; abductive reasoning has been used to diagnose unknown attacks [28]; and ontology- and rule-driven agents have been applied to fault diagnosis in smart homes and building HVAC systems [29,30]. A parallel line relies on large-language-model reasoning, of which Sasha [31] is the prominent example, demonstrating goal-oriented reasoning without structured causal guarantees. *Open challenge:* these methods identify or rank causes but do not return a *calibrated* probability that a proposed intervention will succeed.

### 2.4. Counterfactual Explanations for Time Series

Explanation methods for HAR converge on a consistent finding: residents prefer actionable, cause-sensitive explanations over feature-attribution maps [11,32,33,34,35]. Counterfactual time-series methods emerged in response: CounTS [36] introduces variational counterfactual prediction for time series, and a 2024 survey [35] reviews and benchmarks methods for finding counterfactual explanations. *Open challenge:* this literature largely leaves calibration implicit—how reliable is the predicted intervention probability that a user is expected to act upon?

### 2.5. Probability Calibration

Calibration is a mature subject in its own right: modern classifiers are systematically over-confident [37], and post hoc correction by Platt scaling [38] or isotonic regression [39], assessed with the Brier score (a strictly proper scoring rule [40,41]) or the expected calibration error, is standard practice in the broader machine-learning literature. *Open challenge:* this machinery is rarely brought to bear on smart-home intervention recommendation, where the quantity a resident acts on is precisely a success probability and a well-ranked but poorly calibrated recommendation can be worse than none. Taking that question seriously motivates the Brier-skill and expected-calibration-error assessments of Section 5.3 and Section 5.5.

*Positioning.* Our framework is complementary to all of the above: it pairs ontology-guided semantic normalization with factor-graph counterfactual inference, and it adds a calibrated counterfactual layer—validated under controlled and simulated conditions—that its closest neighbours do not provide.

## 3. Materials and Methods

**Figure 1:**
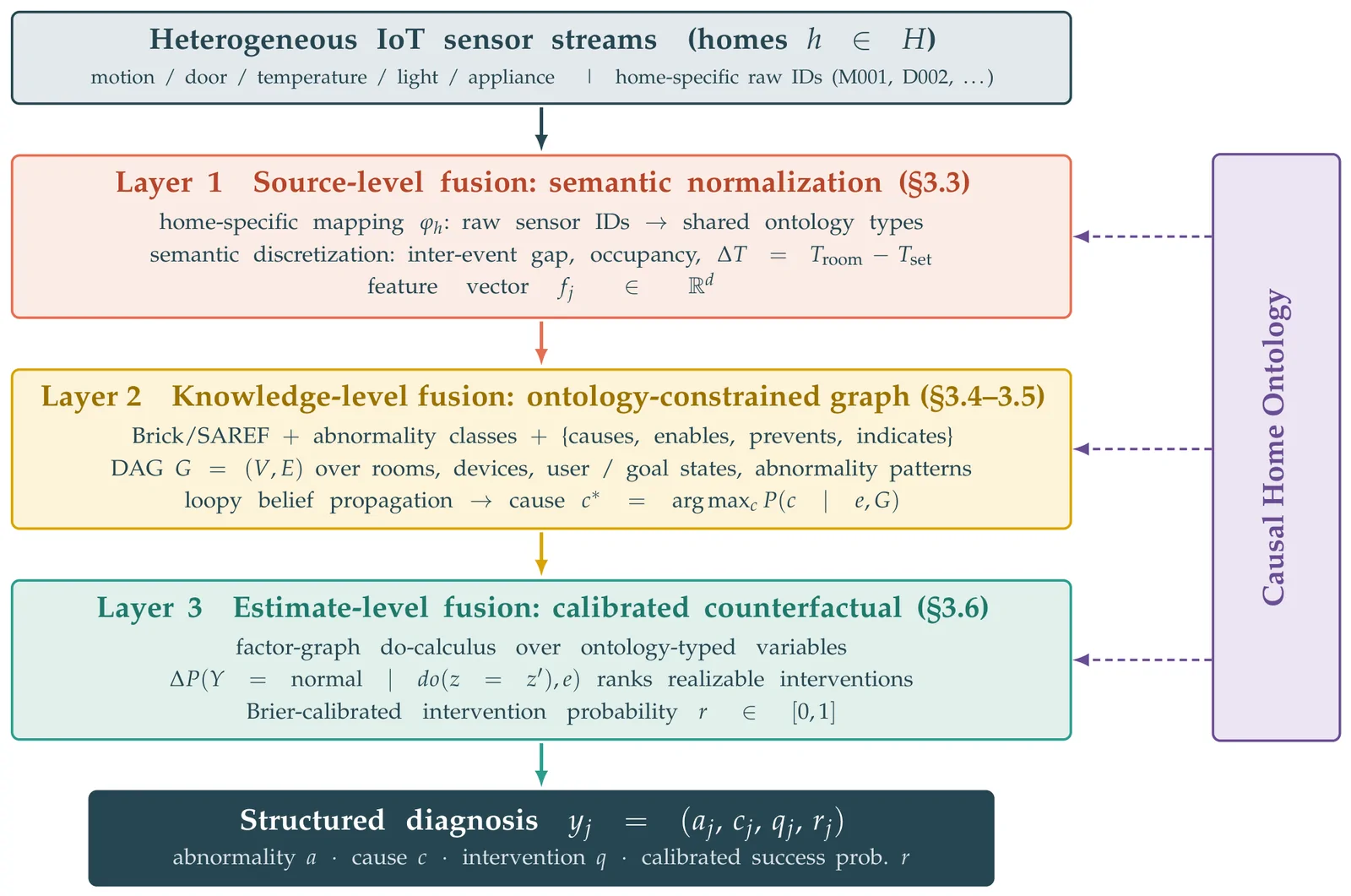
Three-layer ontology-guided information-fusion framework. *Note.* Heterogeneous IoT sensor streams are normalized to a shared ontology vocabulary (source-level fusion), reasoned over by an ontology-constrained causal graph (knowledge-level fusion), and resolved into a calibrated counterfactual intervention probability (estimate-level fusion). The causal home ontology constrains all three layers, and raw device identifiers never leave the first layer. Abbreviations used in the figure: DAG, directed acyclic graph; *h*, home; ID, sensor identifier; IoT, Internet of Things; SAREF, Smart Applications REFerence ontology.

Figure 1 gives the overall picture. The framework is organized as three fusion layers: a source-level layer that normalizes heterogeneous sensor streams into a shared ontology vocabulary, a knowledge-level layer that performs inference over an ontology-constrained causal graph, and an estimate-level layer that produces a calibrated counterfactual intervention probability. The subsections below describe each component and the theoretical analysis that accompanies them.

### 3.1. Problem Formulation

Each home h∈H is instrumented with motion sensors, door (contact) sensors, temperature sensors, and a subset of light and switch sensors. The inventory is heterogeneous across homes, and so are the identifiers: a motion sensor labeled M001 in hh101 may have no counterpart in hh107. Each home therefore produces a stream of raw observations $X_{h}=\left\{x_{i}^{h}\right\}$ over a home-specific symbol set, and we treat the input not as a fixed schema but as a configurable vocabulary that varies per home, with the ontology supplying the shared semantic layer that ties the vocabularies together.

Events are grouped into fixed-length windows. For a window length of k=15 events and a stride of s=15 events, episode *j* comprises $e_{t_{j}}$ through $e_{t_{j}+k-1}$. (The reproducible benchmark-validity audit of Section 5.1 uses a simpler 50-event episode unit with count-based features; the richer windowed featurization described here is part of the framework design but is not all exercised by that audit.) Each episode is summarized by a feature vector $f_{j}\in R^{d}$. It is important to separate what the design specifies from what this study actually computes, because only the latter carries the empirical results. *What is computed and released:* the benchmark-validity audit uses d=16 count-based features per 50-event episode: six per-room event counts (Bedroom, Kitchen, LivingRoom, Bathroom, WorkArea, OutsideDoor), seven episode-level counters (total events, distinct locations, ON, OFF, OPEN and CLOSE transitions, and episode duration in seconds), and the three proxy-rule signature features whose removal defines the rule-blind regime (a night-door count, a 2.0 s burst count, and an away-motion count). Every feature is a plain count or duration over the episode; the construction is fully specified by src/casas_leakage_experiment.py. *What the design specifies but this study does not exercise:* the framework as designed also foresees time-of-day bucketing (night = 0–5 h, day = 6–17 h, evening = 18–23 h), cross-event flags such as window_open_and_cooling_on, and ontology-derived structural features such as room-membership consistency and device-state co-occurrence indicators. These belong to the proposed architecture: they are *not* implemented in the released pipeline and contribute to none of the reported numbers. We therefore state no feature dimensionality for that richer featurization, and the shorter k=15 window is likewise a design parameter rather than a setting used here.

For each episode *j*, the framework predicts a structured tuple $y_{j}=\left(a_{j},c_{j},q_{j},r_{j}\right)$, whose symbols are collected in Table 1, where $a_{j}$ is the abnormality label (Table 2), $c_{j}$ is the most likely cause node in the causal graph, $q_{j}$ is the recommended counterfactual intervention, and $r_{j}\in \left[0,1\right]$ is the calibrated probability that the intervention resolves the situation. The three symbolic components are produced by the released ontology engine in a fixed order, and the engine may decline to emit a tuple at all. It first maps raw evidence to ontology terms and abstains if the mapping confidence falls below 0.5. It then ranks abnormalities against the active evidence and takes $a_{j}$ as the top-ranked one, abstaining if no abnormality is indicated or if the margin between the top two falls below a fixed threshold. Given $a_{j}$, it ranks candidate causes over the relations of Section 3.4 and takes $c_{j}$ as the top-ranked cause, abstaining on an exact tie. Finally, $q_{j}$ is drawn from the interventions the ontology marks admissible for $c_{j}$ (those whose targetsCause is $c_{j}$ and whose requiresEvidence is satisfied), abstaining if that set is empty. Abstention is a deliberate output: the engine reports that it cannot decide rather than returning an unsupported action. The fourth component $r_{j}$ is *not* produced by this engine. It comes from the factor-graph counterfactual module (Section 5.3), which is validated on a controlled structural causal model and on the LBNL FCU command point, so a complete four-component tuple on real home streams is not something this study demonstrates end to end. Three proxy abnormality classes are retained in the label space—AbnormalDoorEvent, SensorDegradation, and OccupancyModeConflict (Section 4.3)—but only SensorDegradation and OccupancyModeConflict have sufficient support for substantive evaluation. The selection rule is stated in advance and is purely a support criterion, not a performance criterion: a class is evaluated only if the raw hh101–hh110 streams contain the instrumentation its proxy rule requires, and it is reported as evaluable only if it reaches at least 100 episodes in the pooled data. ApplianceInconsistency fails the first condition (no appliance-level metering in these homes). AbnormalDoorEvent passes the first but fails the second, with only 3 episodes in 45,969; it is retained in the label space but excluded from the primary three-class macro-F1, because three episodes cannot be reliably evaluated, and it enters only the four-class sensitivity analysis, from which no conclusion is drawn. Applying the threshold before seeing any classifier output prevents the criterion from being tuned to the result.

### 3.2. Causal Home Ontology

The ontology extends Brick [12] and SAREF [13] along three lines those models do not cover: an explicit set of abnormality-pattern classes; four diagnosis-oriented causal relation types (causes, enables, prevents, indicates); and a clean separation between intrinsic device semantics and home-specific configuration. The former describe what a sensor is; the latter describe how it is deployed in a particular home, which is where most cross-home heterogeneity lives. The released OWL/SHACL serialization (CHFD v0.3.0) is the normative specification and contains 32 classes and 13 object properties: 12 core classes aligned to SOSA, Brick and SAREF by rdfs:subClassOf (Home, Room, Sensor, Device, Observation, DeviceState, Evidence, UserState, OperationMode, Abnormality, Cause, Intervention); 7 sensor and device subtypes; 4 abnormality-pattern classes (CoolingLoss, AbnormalDoorEvent, SensorDegradation, OccupancyModeConflict); 5 cause classes; and 4 intervention classes. The full listing is given in Appendix A and the file itself is released. It is machine-checkable, carries reasoner-consistency results and competency questions, and is evaluated under controlled fault injection, while its validation on real streams remains future work (see the scope note in Section 6.3 and Data Availability). Table 3 compares the proposed ontology against Brick, SAREF, DogOnt, and the SSN/SOSA family on the dimensions that matter for diagnosis.

**Table 2:** Proxy abnormality classes retained in the label space (ApplianceInconsistency excluded, Section 4.3).

Class	Description	Primary Signals	Intervention
AbnormalDoorEvent	Door activity inconsistent with the expected home context	Door events, unusual timing, access-sequence anomaly	Inspect entry; verify access
SensorDegradation	Sensor firing is stuck, bursty, or cross-sensor inconsistent	Burst patterns (≥3 events within 3 s), flat signal, cross-sensor mismatch	Recalibrate or replace
OccupancyModeConflict	Occupancy detected during an away or secured context	Motion during Leave_Home → Enter_Home spans, mode mismatch	Reconcile occupancy/security mode

*How the ratings in Table 3 were assigned:* each cell records whether a model provides the vocabulary needed for one capability of *this* diagnostic task; it is not a judgement of the model’s overall quality, and each of these ontologies is well-designed for the purpose it was built for. We inspected the released vocabularies cited in Section 2 and applied a fixed rubric. A capability is marked ✓ (supported) when the model defines a named class or property whose intended semantics express it directly and whose use is constrained by axioms (a declared domain and range, or a subclass axiom), so that a reasoner can check it. It is marked Partial when the capability can be expressed only indirectly, through a generic property or a free-text annotation, or only for a subset of the cases we need. It is marked Weak when a term of the right name exists but carries no constraining axioms, so its use cannot be machine-checked. It is marked Obs. (observation-only) when the model can record the underlying measurement but offers no typed relation for the diagnostic role that measurement plays. It is marked × when no corresponding term exists. Ratings were fixed before the ontology prototype was evaluated.

**Table 3:** Comparison with existing smart-home semantic models. ✓ = supported; Partial = partially supported; Weak = present but under-specified; Obs. = observation-only; × = not supported.

Capability	Brick	SAREF	DogOnt	SSN	Ours
Device/sensor semantics	✓	✓	✓	✓	✓
Room/location context	✓	Partial	Partial	×	✓
User-state modeling	×	×	Partial	×	✓
Abnormality patterns	×	×	×	×	✓
causes relation	Weak	Weak	Weak	Obs.	✓
enables/prevents	×	×	×	×	✓
indicates relation	×	×	×	Obs.	✓
Counterfactual support	×	×	×	×	✓
Cross-home mapping	×	×	×	×	✓

*Note.* For *Ours*, counterfactual support and cross-home mapping are demonstrated at the ontology-vocabulary level and in the controlled prototype (Appendix A), not validated on real home streams—a specified and prototyped capability, not a deployed one.

### 3.3. Semantic Discretization

Converting heterogeneous event streams into discrete semantic states is the first preprocessing step. The inter-event gap Δt is bucketed into eight bins (0–10 s, 10–30 s, 30 s–1 min, 1–5 min, 5–30 min, 30 min–2 h, 2–8 h, 8 h+), and absolute time of day into the three regimes given above. Cooling and heating setpoint deviation is expressed relatively as $\Delta T=T_{\mathrm{room}}-T_{\mathrm{setpoint}}$ and discretized with the threshold τ=μ±1.5σ computed on a per-home baseline period; expressing the deviation relative to a local baseline avoids the well-known pitfall of absolute thresholds that travel poorly across climates. Window and door open-durations are discretized symbolically (Brief <1 min, Sustained 1–10 min, Extended >10 min). The complete set of variables, bin boundaries, and home-specific calibration rules appears in Table 4.

### 3.4. Ontology-Constrained Causal Graph

For each diagnosis episode, the causal graph G=(V,E) is instantiated as a directed acyclic structure [42,43]. Its vertices fall into six ontology-typed groups (rooms (*R*), devices (*D*), user states (*U*), goal states (*Q*), abnormality patterns (*A*), and exogenous context (*X*)) and its edges are constrained by the four ontology relation types, inheriting their semantics. The causes relation is the diagnostic backbone, connecting a cause node to its abnormality pattern; enables encodes a precondition (an open window enables cooling-loss); prevents marks a blocking condition (a sealed window prevents draft-induced cooling-loss); and indicates marks an observable that is correlated with, but not causally linked to, an abnormality. Temporal unrolling within an episode preserves the DAG property by indexing repeated visits to the same logical node with a time superscript, so a single physical device can appear at several time slices without inducing a cycle.

*Construction procedure, and what is implemented:* the released engine (ontology_prototype/src/ontology_engine.py) instantiates the graph for an episode directly from the ontology, with no learned structure. Given a diagnosed abnormality *a*, it issues one SPARQL query per relation type and adds, for each triple 〈s,ρ,a〉 with ρ∈{causes,enables,indicates,prevents}, a vertex *s* typed by ρ and a directed edge s→a carrying ρ as its label. The vertex set is therefore exactly the set of ontology individuals standing in one of the four relations to *a*, and the edge set is exactly the corresponding triples: both are determined by the ontology file, so the graph for a given abnormality is identical on every run and on every home. Candidate causes are then scored against the active evidence, and an intervention is admissible only if its targetsCause is the selected cause and its requiresEvidence set is contained in the active evidence.

Two elements of the design above are *not* exercised by this implementation, and we flag them explicitly so the description is not read as a report of working code. The graph built by the released engine is a single-layer, one-hop structure around the diagnosed abnormality: there is no temporal unrolling, so the time-superscript construction is a design provision rather than something the prototype performs. Nor does the engine hold conditional probability tables; cause ranking is a deterministic evidence-matching score over ontology relations, and the calibrated probabilities reported in this paper come from the separate factor-graph module of Section 5.3, which is evaluated on a controlled structural causal model rather than on an ontology-instantiated episode graph. Specifying and fitting per-episode conditional probability tables on real streams is exactly the step we scope as future work.

### 3.5. Structural Diagnosis Inference

Diagnosis selects $c^{*}=\arg\max_{c\in C}P\left(c|e,G\right)$, the most probable cause given the evidence *e* and the graph *G*. Although the per-episode causal model is acyclic, the factor graph used for inference is not: moralization of the causal graph and the sharing of ontology-relation hub variables introduce loops, so exact belief propagation does not apply. The framework therefore uses loopy belief propagation (LBP) as an approximate MAP solver, and the acyclicity of the causal model should not be confused with acyclicity of the inference graph. As a concrete example, suppose OpenWindow and ExcessiveCoolingDemand both cause CoolingLoss and both are indicated by the same room-temperature reading. The causal graph is acyclic—both edges point into CoolingLoss—but moralization marries the two parents (they share a child) and links both to the shared temperature evidence, closing the loop OpenWindow–CoolingLoss–ExcessiveCoolingDemand–temperature–OpenWindow. Exact belief propagation is then inapplicable to the inference graph even though the causal model has no cycle, which is precisely why damped LBP is used. Damped message updates (α=0.5) prevent the oscillations that ontology-relation hubs would otherwise induce in cycles. Convergence is declared once the maximum message change drops below $\varepsilon =10^{-5}$, or after the iteration cap of T=300 is reached. Both values are the ones used in the convergence characterization of Appendix B; the cap is set well above the observed median iteration count so that it does not truncate runs that would otherwise converge. By design, the per-episode cost is O(T|E|) in the number of ontology-induced edges |E| and the iteration cap *T*, so inference is intended to be linear in the local graph size. Appendix B reports an initial convergence characterization on synthetic factor graphs representative of the causal structure: in the moderate-coupling regime that matches the diagnostic potentials, damped LBP converges in 100% of runs across graph sizes and initializations (median 40–104 iterations; moderate damping α∈[0.25,0.5] is fastest, consistent with the default α=0.5), whereas strong couplings oscillate at all damping levels, a known LBP limitation. The end-to-end run on *real streams* remains part of the implementation gap (Section 6.3). The prior calibration, the evidence-bucket schedule, and the tie-breaking rules are summarized in Table 5.

### 3.6. Counterfactual Explanation

For each predicted abnormality, the framework evaluates a counterfactual intervention $\mathrm{do}(z=z^{\prime })$ and ranks the candidates by $\Delta P(Y=\mathrm{normal}|\mathrm{do}\left(z=z^{\prime }\right),e)$, following Wachter et al. [32]. The intervention space is restricted to actions a resident or building operator can realistically perform: setting a device state (e.g., closing a window or raising a setpoint), correcting an occupancy-mode toggle, or invalidating a degraded sensor. The factor-graph estimator returns calibrated probabilities, and we report two acceptance thresholds: r≥0.70 for routine recommendations and r≥0.85 for high-confidence automated interventions. These thresholds are illustrative operating points selected for the controlled evaluation; they are not theoretically guaranteed and require application-specific validation before any deployment use.

As a concrete illustration, consider an episode in which a window-open event co-occurs with the cooling system running (the window_open_and_cooling_on flag). The causal graph attributes the resulting cooling-loss abnormality to the open window through a causes edge, and the counterfactual stage evaluates do(window=closed), which removes the enables precondition. If the calibrated success probability clears the 0.85 threshold, the action is issued automatically; otherwise, it is surfaced as a routine recommendation. The same machinery declines to act on a merely indicates correlate (say, a motion sensor that happens to fire nearby) because no prevents edge connects it to the abnormality, so it is never proposed as an intervention. Algorithm 1 specifies the intended end-to-end procedure; it is *not* executed as a single pipeline in the present experiments, which evaluate its components separately.

## 4. Experiments

The five evaluations in this section use different data, labels, and protocols, and they establish different things. To keep those distinctions explicit—and to make clear which results come from the ontology-guided pipeline and which from conventional tabular classifiers—Table 6 summarizes the components of each evaluation and Table 7 states what each does and does not establish. The reproducible CASAS and HomeC audits and the LBNL classification use conventional feature-fed classifiers; only the SCM counterfactual layer and the ontology prototype exercise the framework’s structured components, and both run under controlled or synthetic conditions.

### 4.1. Dataset and Home Selection

The evaluation uses ten homes from the CASAS smart-home dataset [2,18,19], chosen because each contains enough event volume to support windowed inference without sparsity artifacts. The protocol is leave-one-home-out: at each fold, one home is held out for testing while the remaining nine supply training and calibration data, and the procedure rotates through all ten homes. In the reproducible audit (Section 5.1), this yields $n_{\mathrm{eff}}=$ 15,435 positive test episodes. Table 8 summarizes the datasets and the home selection.

### 4.2. Data Preprocessing and Feature Construction

Every reported number is produced from public raw data through a documented raw-to-feature pipeline; the three preprocessing modules (preprocess_casas.py, preprocess_lbnl.py, and preprocess_homec.py) are released with the code and specified in full in the repository’s PREPROCESSING.md, and each runs a self-test on synthetic input so the pipeline is executable without the third-party data.

For CASAS, the raw date,time,place,state,act event rows are parsed into a chronologically ordered stream, per-event signatures are derived (outside-door openings, ON/OFF and open/close transitions, night-time door events, near-uniform 2.0 s same-location bursts, and motion within a Leave_Home→Enter_Home span), and the stream is partitioned into non-overlapping 50-event episodes. Each episode yields thirteen generic count features (per-room event counts, total event and unique-location counts, ON/OFF and open/close counts, and episode duration) together with three rule-signature features; the proxy labels of Section 4.3 are attached at this stage, and the resulting feature table is byte-identical to the released casas_windows.csv.

For the LBNL FDD Fan Coil Unit data, the fifteen fault-scenario files are mapped to seven fault classes, the twenty-nine simulated channels are coerced to numeric and cleaned, and seven physics-based features are derived (cooling and heating setpoint deltas, cooling/heating valve position-versus-command and damper mismatches, and discharge-versus-return and mixed-versus-outdoor air-temperature differences); within each scenario, the first 70% of time steps form the training split and the last 30% the test split, so no future information leaks into the past.

For HomeC, the 1 Hz appliance stream is aggregated into five-minute episodes (per-appliance means and house-level mean, maximum, and standard deviation), labeled by published physical thresholds (the LBNL One-Watt standby band and an NREL two-sigma energy-spike criterion), and split temporally; because HomeC is a single home, this is a temporal rather than a cross-home split; we fit the labelling thresholds on the training segment only (the --leakage-free path), and the audit of Section 5.4 shows the resulting labels remain recoverable from the power features.

### 4.3. Proxy Anomaly Construction

The hh101–hh110 streams carry no expert fault annotations, so we define three anomaly classes by explicit, disclosed proxy rules applied directly to the raw events; the rules, not the dataset, are the labels, and they are released with the code, so the audit of Section 5.1 is fully reproducible. AbnormalDoorEvent marks outside-door open events at clock hours h∈{2,3} a.m.; in the raw streams, such night-time openings are extremely rare (three 50-event episodes across the ten homes), so this class is effectively unpopulated and we do not evaluate it. SensorDegradation marks same-sensor bursts at a near-uniform 2.0 s inter-event interval and is the most populated class (14,575 episodes); the microsecond timestamps within a burst often share a common offset, a clear algorithmic signature. OccupancyModeConflict marks motion during a Leave_Home→Enter_Home span (857 episodes). ApplianceInconsistency is excluded for the reasons in Section 4.3 (sparse appliance-level coverage). Because every rule leaves a deterministic temporal signature, a classifier given features that encode those signatures can recover the labels almost perfectly without diagnosing any real fault; Section 5.1 exactly measures this leakage. The protocol therefore probes cross-home semantic transfer under controlled anomaly generation, not real-world detection under expert annotation. The episode counts above are reproduced by the released pipeline; earlier event-level counts that could not be regenerated from the raw streams have been removed.

Because the proxy classes are defined by deterministic, disclosed rules rather than expert annotation, the appropriate reliability question is how much of the achievable score is rule leakage rather than diagnosis; the benchmark-validity audit (Section 5.1) answers this directly. We do not report a separate inter-rater study: an earlier annotation exercise could not be regenerated from the raw streams (in particular, the AbnormalDoorEvent class is near-absent there; Section 5.1), so its agreement figures are withdrawn.

*Why ApplianceInconsistency is excluded.*

The proxy rule in Table 9 specifies that appliance and item events inconsistent with the surrounding activity context should be labeled, but three properties of the hh101–hh110 deployment make this rule unworkable as a benchmark target. First, item-type events make up under 5% of all observations, and the dataset uses a generic item identifier rather than appliance-specific labels, so a microwave cannot be distinguished from a television or a computer power state. Second, the class is too sparse to annotate or evaluate reliably on these homes. Third, the activity-label alignment that any appliance/activity-inconsistency rule depends on is noisy in CASAS, with overlapping labels and multi-resident segments in several homes. We treat appliance-fault diagnosis as a separate problem that requires richer instrumentation: smart-plug power monitoring or appliance-specific sensor IDs, which are available in REDD [44] or AMPds2 [45] but not here. Section 6 names this as the natural next benchmark.

### 4.4. Baselines and Audit Protocol

Because the benchmark-validity audit (Section 5.1) replaces architecture-versus-representation comparisons with a controlled, shared-feature design, our baselines are three model families trained on identical inputs: gradient-boosted trees (GBT, implemented as scikit-learn’s HistGradientBoostingClassifier) [46], a random forest (RF), and logistic regression (LR). We use GBT, RF and LR from this point on. The three were chosen to span the inductive biases that matter for this audit while keeping the comparison interpretable: GBT is the strongest general-purpose learner for small, heterogeneous tabular feature tables of this kind and is the estimator used throughout; RF is a bagged, high-variance-reduction alternative whose failure modes differ from boosting, so agreement between the two is evidence about the representation rather than about one optimizer; and LR is a linear model with no capacity to synthesize interactions, so if a proxy rule is recoverable even linearly, the leakage is in the features themselves and not in model expressiveness. Deep sequence models were deliberately excluded: they consume a different input (raw event sequences), which would reintroduce exactly the representation-versus-architecture confound the shared-feature design removes. Running the three on the same feature set isolates the effect of the representation from the effect of model capacity, which a comparison between feature-fed trees and raw-sequence neural networks cannot do. Every model receives the same feature set on each benchmark, so the comparison turns on representation rather than architecture. Table 10 summarizes the configurations; measured performance is reported in Section 5.1, Section 5.2 (CASAS audit), Section 5.4 (HomeC), and Section 5.5 (LBNL FDD).

*Software environment and exact configurations:* all experiments run on Python 3.10 with scikit-learn 1.7.2, XGBoost 3.2.0, NumPy 2.2.6, pandas 2.3.3, and SciPy 1.15.3 (pinned in environment-lock.txt, with data and output checksums in CHECKSUMS.txt); every model uses a fixed random seed of 42, and no result depends on unseeded randomness. The estimator hyperparameters differ by benchmark and are exactly those in the released scripts; Table 11 lists them in one place. The released repository is archived at a tagged release (cited in the Data Availability statement), so every reported number regenerates from that fixed revision; the single command python check_all.py re-runs each source experiment and checks its output against the values printed in this paper.

### 4.5. Evaluation Metrics

We report macro-F1 and per-class F1 for diagnosis and, for the counterfactual layer, the Brier score, Brier skill score, and expected calibration error (ECE) [37]. On the CASAS audit, we add a permuted-label control as a chance floor. We do not report a Top-1 cause-attribution accuracy on the proxy benchmark: because the candidate causes are derived from the same proxy rules that define the labels, such a number would be circular (Section 5.1).

### 4.6. Statistical Analysis

Uncertainty on the CASAS audit is quantified by leave-one-home-out folds (ten homes) and a permuted-label control. Because windows within a home are not independent, confidence statements use home-level (cluster) resampling rather than episode-level bootstrap. On the synthetic SCM and the LBNL FDD benchmark, confidence intervals use bootstrap resampling over the held-out set. We avoid the pairwise significance claims of the original draft, which depended on the now-withdrawn accuracy comparison against raw-sequence baselines.

## 5. Results

### 5.1. Benchmark-Validity Audit: Proxy-Label Leakage on CASAS

Because the CASAS hh101–hh110 anomalies are defined by the disclosed proxy rules of Section 4.3 rather than by expert annotation, a high cross-home F1 on this benchmark can mean two very different things: genuine semantic transfer, or a classifier that has rediscovered the rules used to generate the labels. We test which one operates, using a fully reproducible pipeline released with the paper. We rebuild the three proxy classes directly from the raw event streams (AbnormalDoorEvent: outside-door open at 2–3 h; SensorDegradation: same-sensor bursts at a ≈2.0 s inter-event gap; OccupancyModeConflict: motion during a Leave_Home→Enter_Home span), cut each home’s stream into 50-event episodes, and evaluate under leave-one-home-out cross-validation (45,969 episodes over the ten homes, of which 15,435 are positive (non-Normal)). On these raw streams, the AbnormalDoorEvent class is essentially absent (three episodes in total, because night-time outside-door openings almost never occur), so the audit is informative for SensorDegradation and OccupancyModeConflict; this absence is a finding in its own right, since it shows that the door-anomaly statistics quoted in some prior treatments of this benchmark cannot be recovered from the raw data.

**Figure 2:**
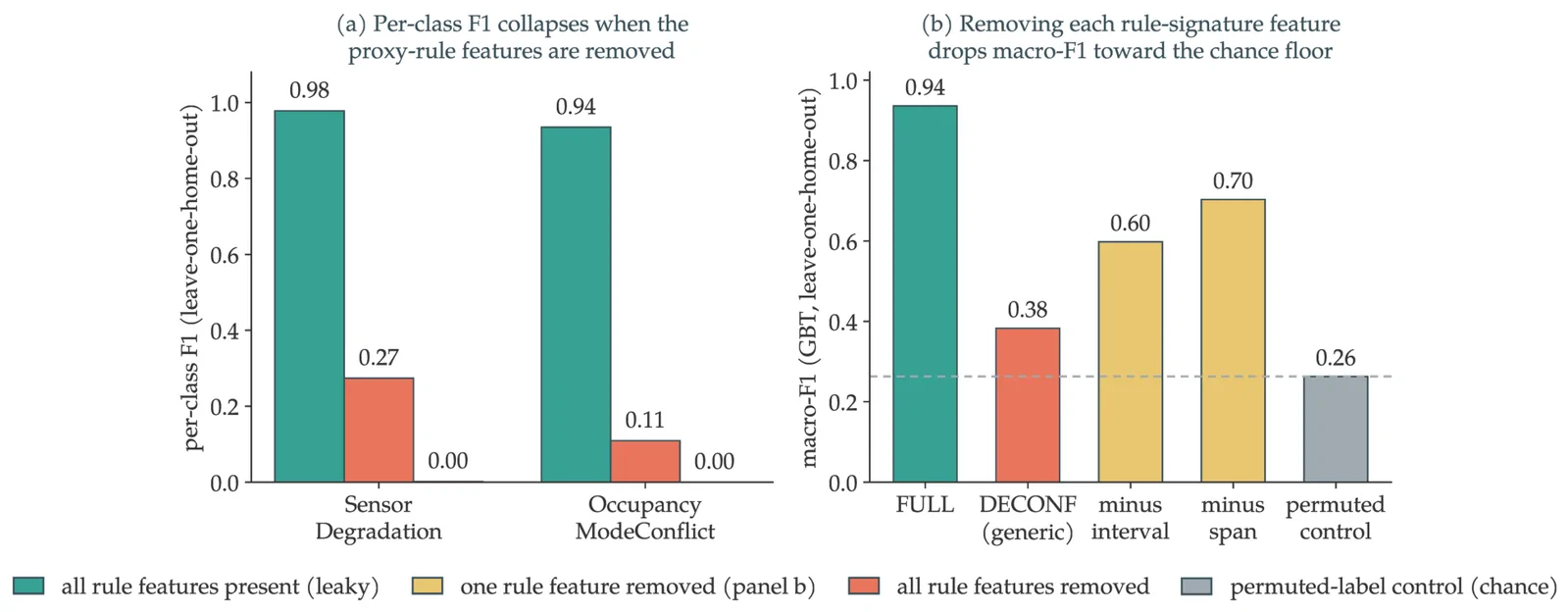
CASAS proxy-label leakage audit. *Note.* (**a**) Per-class F1 collapses when the proxy-rule features are removed. (**b**) Leave-one-rule-out: each rule-signature feature drops macro-F1 toward the chance floor. Leave-one-home-out cross-validation.

We compare two feature regimes. The rule-exposed regime adds three features that re-encode the proxy rules (a night-door count, a 2.0 s-burst count, and an away-motion count) to a set of generic semantic features (per-room event counts, on/off and open/close counts, episode duration). The rule-blind regime keeps only the generic features. Table 12 and Figure 2 report the result, and the pattern is clear. With the rule features present, GBT reaches macro F1 0.936 (primary, over Normal and the two supported anomaly classes), with per-class F1 of 0.978 (SensorDegradation) and 0.935 (OccupancyModeConflict). Removing them drops macro F1 to 0.383 and per-class F1 to 0.274 and 0.109, only 0.120 above the permuted-label floor of 0.263. The leave-one-rule-out rows localize the effect: dropping the 2.0 s burst feature alone collapses SensorDegradation (from 0.978 to 0.282), and dropping the away-motion feature alone collapses OccupancyModeConflict (from 0.935 to 0.154). The per-class scores are thus carried almost entirely by the features that re-encode each class’s generation rule.

*Which classes enter the macro average:* the primary macro-F1 in Table 12 is averaged over the three classes with usable support—Normal, SensorDegradation and OccupancyModeConflict. AbnormalDoorEvent (an outside-door opening at 02:00–03:00) occurs in just 3 of the 45,969 episodes in the raw hh101–hh110 streams, so it carries no usable support; we exclude it from the primary metric and report it separately (Section 4.3), so the audit is effectively over two anomaly classes plus Normal. For transparency, the released pipeline also computes a four-class macro-F1 that includes the degenerate class as a sensitivity value: it is uniformly lower (rule-exposed 0.702, rule-blind 0.287, permuted floor 0.197) because the near-empty class contributes an F1 of approximately zero, but the collapse and every audit conclusion are identical under either metric.

**Table 12:** Benchmark-validity audit on CASAS hh101–hh110 (GBT; leave-one-home-out CV; 50-event episodes; primary macro-F1 over Normal and the two supported anomaly classes, and per-class F1).

Feature Configuration	Macro F1	SensorDegr. F1	OccupancyConflict F1	#Feat.
Rule-exposed (generic + 3 rule features)	**0.936**	0.978	0.935	16
Rule-blind (generic only)	0.383	0.274	0.109	13
drop 2.0 s-burst feature	0.598	0.282	0.802	15
drop away-motion feature	0.703	0.992	0.154	15
Permuted-label control	0.263	0.002	0.000	16

*Note.* Per-class scores collapse when the proxy-rule features are removed, and the leave-one-rule-out rows show that each class is carried by the feature that re-encodes its generation rule. AbnormalDoorEvent is omitted from the per-class columns (three episodes total in the raw streams). All values are measured; the pipeline is released.

*The leakage is directionally consistent across all ten homes.* Under leave-one-home-out, every held-out home shows a positive rule-exposed→rule-blind drop (Table 13): the drop ranges from 0.298 (hh103) to 0.671 (hh107), with mean 0.552 [0.482, 0.606] (bootstrap over the ten homes). The rule-exposed score varies across homes (median 0.967, IQR [0.941,0.989]) while the rule-blind score is uniformly near the permuted-label floor (median 0.385). This is directional consistency, not identical effect magnitude: the leakage is a property of the proxy-rule labelling that recurs in every home, not an artifact of one or two homes.

*Per-home and per-class composition.* Table 14 gives the episode counts per home and per class that underlie the audit, so the class balance and the sparsity of AbnormalDoorEvent are visible directly. Across the ten homes, the pooled prevalence is Normal 66.4%, SensorDegradation 31.7%, OccupancyModeConflict 1.9%, and AbnormalDoorEvent 0.006% (three episodes total). The Normal class dominates every home, which is why we report macro-F1 rather than accuracy, and OccupancyModeConflict is entirely absent from hh103, so its per-home score there is undefined and is excluded from that home’s macro average.

### 5.2. Fair Baselines Under a Shared Feature Set

The original draft of this work compared a feature-fed GBT model against sequence baselines (Long Short-Term Memory, Transformer) that received raw event streams, and reported a large gap. That comparison confounds model architecture with input representation, so we replace it with a controlled one: the same feature set for every model. Table 15 runs GBT, a random forest, and logistic regression on identical inputs. Under the rule-blind features, all three land within 0.03 of one another (0.383, 0.36, 0.383); under the rule-exposed features, all three are high (0.936, 0.967, 0.921). The model family is therefore not the deciding factor on this benchmark; the feature representation is. This reframes, rather than contradicts, the original ablation finding that “semantic normalization, not model capacity, drives the score”: the decisive signal on CASAS is largely the proxy-rule encoding, a property of the benchmark rather than of the framework.

**Table 13:** Per-home CASAS benchmark-validity audit (leave-one-home-out; macro-F1; GBT).

Held-Out Home	Rule-Exposed	Rule-Blind	Drop
hh101	0.960	0.390	0.570
hh102	0.941	0.454	0.487
hh103	0.666	0.368	0.298
hh104	0.909	0.414	0.495
hh105	0.991	0.401	0.590
hh106	0.941	0.403	0.538
hh107	0.991	0.320	0.671
hh108	1.000	0.371	0.629
hh109	0.982	0.379	0.603
hh110	0.974	0.333	0.641
Mean [95% CI]	0.935 [0.868, 0.978]	0.383 [0.361, 0.407]	0.552 [0.482, 0.606]
Median [IQR]	0.967 [0.941, 0.989]	0.385 [0.369, 0.403]	0.580 [0.506, 0.623]

*Note.* Every home shows a positive rule-exposed−rule-blind drop, so the leakage is directionally consistent across all ten homes rather than driven by a subset; aggregate bootstrap is over the ten homes.

**Table 14:** CASAS hh101–hh110 episode counts per home and per class.

Home	Total	Normal	AbnormalDoor	SensorDegr.	OccupancyConflict
hh101	4457	2755	0	1560	142
hh102	5637	3542	0	1999	96
hh103	2249	1279	0	970	0
hh104	6894	4813	0	2055	26
hh105	2941	1756	0	1107	78
hh106	4050	2952	0	1034	64
hh107	4065	2467	0	1297	301
hh108	5715	3936	3	1758	18
hh109	7986	5709	0	2153	124
hh110	1975	1325	0	642	8
**Total**	45,969	30,534	3	14,575	857
Prevalence	—	66.4%	0.006%	31.7%	1.9%

*Note.* Non-overlapping 50-event episodes; counts are reproduced by src/casas_leakage_experiment.py. AbnormalDoorEvent appears in only hh108 (3 episodes), so it is retained in the label space for consistency but carries no usable support (Section 5.1).

**Table 15:** Fair-baseline comparison on CASAS under identical feature sets.

Model	Rule-Blind Features	Rule-Exposed Features
GBT	0.383	0.936
Random forest	0.36	0.967
Logistic regression	0.383	0.921

We draw two conclusions from the audit. First, CASAS hh101–hh110 should be used as a semantic-transfer and benchmark-validity probe, not as evidence of fault-detection accuracy; we accordingly report no headline accuracy number for it. Second, the framework’s value must rest on what the audit cannot explain away: the calibrated counterfactual layer (Section 5.3) and the calibration behavior on labeled faults (Section 5.5), to which we now turn.

### 5.3. Controlled SCM Validation: Counterfactual Intervention Calibration

The CASAS audit above concerns disclosed proxy labels, and the proxy protocol cannot speak to the quality of the counterfactual layer: on real data, the true causal effect of an intervention cannot be observed without physical actuation. To validate that layer directly, we conduct a controlled experiment on a synthetic structural causal model (SCM) whose ground-truth intervention effects are known by construction.

The SCM has four observed variables (DoorState, OccupancySignal, SecurityMode, and EventBurst) and one latent variable, SensorHealth. It encodes three anomaly classes (AbnormalDoorEvent, SensorDegradation, OccupancyModeConflict) and three identifiable do-interventions, do(DoorState=0), do(EventBurst=0), and do(OccupancySignal=0). The causal graph satisfies the back-door criterion [47] for all three interventions, so the average treatment effect is identifiable from observational data. We generate ten synthetic homes of 3000 windows each (30,000 windows in total) and evaluate counterfactual reasoning under sensor noise σ=0.4, which simulates cross-home feature shift. The ground-truth intervention removal rates, computed directly from the SCM, are 92.4%, 92.2%, and 92.0% for the three classes, confirming that the interventions are both effective and well-separated.

Two properties of the framework emerge clearly from this controlled setting (Table 16). First, the factor graph returns calibrated estimates of P(normal∣do(X=x),e) for each candidate intervention rather than a single argmax label. Against the SCM ground truth, the Brier score is 0.158 [0.150, 0.165], giving a Brier skill score of 0.369 over the uninformed baseline of 0.250 (p<0.001). This calibration property is specific to the factor-graph formulation. A point-prediction classifier does not natively output an intervention-success probability; to avoid an empty comparison, we add an isotonic-calibrated [39] GBT baseline (Platt scaling [38] gives essentially the same result) that predicts the success probability of its chosen intervention. Even against this calibrated baseline, the factor graph is the better-calibrated forecaster (Brier 0.158 vs. 0.204; Brier skill score 0.369 vs. 0.183), because it reasons over the causal structure rather than mapping noisy features to a probability.

**Figure 3:**
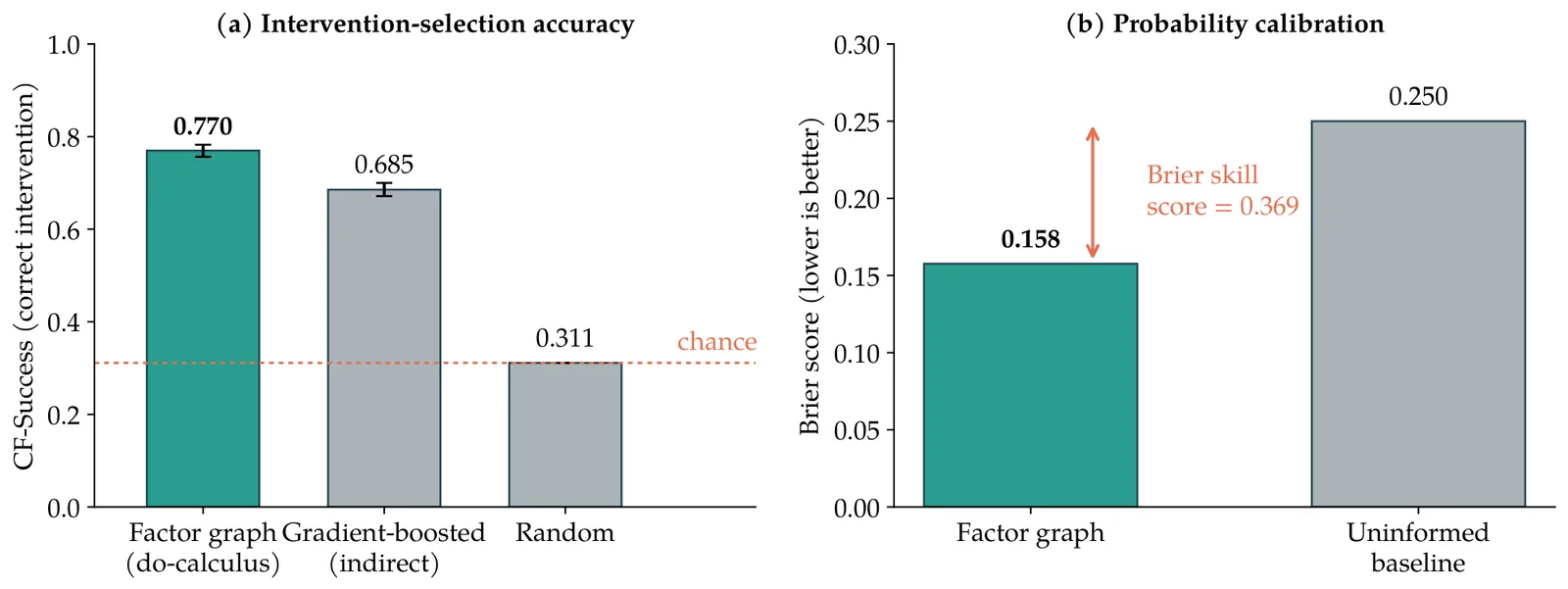
Counterfactual calibration on the controlled SCM. *Note.* σ=0.4. (**a**) The factor graph selects the SCM-oracle-confirmed intervention far more often than indirect selection or chance. (**b**) Its intervention probabilities are well-calibrated (Brier skill score 0.369). Values and baselines are in Table 16.

Second, intervention selection is more accurate: given a noisy anomaly episode, the factor graph with do-calculus inference picks the intervention that the SCM oracle confirms resolves the anomaly on 0.770 [0.756, 0.782] of episodes, against 0.685 [0.672, 0.700] for indirect classifier-based selection and 0.311 for random choice (p<0.001). The reason for the gap is straightforward: a point-prediction classifier chooses an intervention only indirectly, through its predicted anomaly class, so feature noise propagates through the prediction; the factor graph reasons over the causal graph directly, and the noise does not compound. Figure 3 summarizes both effects. All SCM values reported here are regenerated by the released counterfactual emulator (exact do-calculus inference, fixed seed); the emulator is a reference implementation of the controlled SCM specified above.

### 5.4. Extending the Audit to HomeC (UMass Smart*)

We next apply the same benchmark-validity audit to real appliance-level measurements: the HomeC home from the UMass Smart* dataset [48] (the entire released HomeC home: about 503,900 one-second readings over 5.8 days across eleven appliance power channels plus house-total power and weather; we use all of it, not a subset). Following the preprocessing of Section 4.2, we aggregate to five-minute episodes (1680 episodes) and label them by published physical thresholds: StandbyPhantomLoad from the LBNL One-Watt standby band (>5 W idle draw) and EnergyAnomaly from the NREL 2σ building-performance rule. In contrast to the exploratory run in an earlier version of this work, we fit all labelling thresholds on the training segment only (the released --leakage-free path) and use a fixed temporal 70/30 split (seed 42), so the labels never see the test segment.

The result (Table 17) mirrors CASAS. A GBT on the full thirteen-feature set reaches macro-F1 0.963 [0.833, 1.000]; but the physical thresholds are computed from four of those same features (house-total mean and maximum, wine-cellar mean, home-office mean), so removing that group collapses macro-F1 to 0.445 [0.330, 0.585], with a permuted-label control at 0.355 [0.316, 0.407]. The residual gap between the rule-blind model and the permuted control is 0.079 with a bootstrap confidence interval of [−0.061,0.242] that *includes zero*; we therefore read the rule-blind score as near-floor degradation with no statistically significant residual signal, not as evidence of transferable fault detection. The small test-set counts for the rare classes (19 StandbyPhantomLoad and only 4 EnergyAnomaly test episodes) make the per-class F1 of EnergyAnomaly, and its confidence interval, statistically unstable: a single misclassified test episode shifts that class’s F1 by roughly 0.1–0.2, which is what widens the macro-F1 intervals. We therefore draw *no per-class conclusion* for EnergyAnomaly and rely instead on the macro trend and on the rule-blind−permuted gap, whose zero-spanning interval is stable across the 20 seeds; the four-episode class is reported for completeness, not as evidence.

The collapse is robust and model-independent. Over **20 random seeds** (one fixed temporal split throughout), GBT and eXtreme Gradient Boosting (XGBoost) agree on the rule-exposed score (0.963) and give the same drop; the rule-blind-permuted gap has median 0.120 (IQR [0.106,0.131]) for GBT and 0.128 (IQR [0.115,0.142]) for XGBoost, while its test-set bootstrap 95% CI is [−0.078,0.199]. We fix the interpretation in advance: a gap CI that includes zero denotes a *near-floor, statistically unresolved residual signal*; a CI wholly above zero would denote *limited residual predictive information* (never real-fault validity); and a high rule-exposed score denotes *label-generating-rule recoverability*. HomeC falls in the first and third categories: the small, seed-stable but statistically unresolved rule-blind residual is at most limited predictive information, and the rule-exposed score recovers the labelling rule. A control confirms this is generic rule-recoverability rather than physics: an arbitrary threshold proxy with the same class prevalence but defined on unrelated appliance features (refrigerator and furnace power) is recovered just as well (rule-exposed 0.980, collapsing to 0.553 once its own defining features are removed). The high rule-exposed score therefore reflects recovery of any feature-derived threshold rule, physical or not.

HomeC is thus a second instance of proxy-label circularity, on genuinely real measurements: because the labels are a near-deterministic rule over the observed power features, a high rule-exposed score reflects recovery of the label-generating rule rather than independent fault-detection ability. The distinction from CASAS is instructive: the CASAS proxy rules are somewhat arbitrary temporal signatures, whereas the HomeC labels are *physically principled* thresholds from published standards (LBNL One-Watt standby; NREL 2σ). That even physics-grounded threshold labels are circular, because the threshold variables (Table 18) are themselves inputs to the classifier, shows the leakage is a property of threshold-on-observed-feature labelling in general, not of any one benchmark’s rule design. This reinforces the paper’s central finding that high scores on threshold- or rule-labelled smart-home benchmarks must be audited before they are read as diagnosis, and it means HomeC does not provide independent real-fault validation of the framework. An earlier configuration of this work reported macro-F1 0.656 on HomeC; the released script homec_leakage.py reproduces it exactly. That figure is itself a rule-blind score—it excludes all four threshold-defining features (Table 18)—but is computed over a different feature set from the audit of Table 17: it admits two further appliance channels (barn and dishwasher power) that the audit list omits and drops the house-power variance. The rule-blind macro-F1 is therefore feature-set-dependent, ranging from 0.445 on the audit’s pre-specified set to 0.656 when the two extra appliance channels are included; both remain far below the rule-exposed 0.963. We base the circularity claim on the audit set of Table 17, for which we report the permuted-label control and a rule-blind-permuted gap whose confidence interval includes zero; the 0.656 variant carries no permuted control, so we draw no independent-validation conclusion from it and read it only as a wider-feature rule-blind point most plausibly reflecting indirect correlation of whole-house appliance states with the standby label. Under either rule-blind feature set, HomeC does not provide independent real-fault validation of the framework. The framework’s distinguishing contribution accordingly rests not on HomeC accuracy but on the calibrated counterfactual layer (Section 5.3) and the calibration behaviour on labelled faults (Section 5.5).

**Table 17:** Benchmark-validity audit on HomeC.

Configuration	GBT Macro-F1	XGBoost Macro-F1	#Feat.
Rule-exposed (all features)	**0.963**	**0.963**	13
Rule-blind (drop 4 threshold feats)	0.445	0.460	9
Permuted-label control	0.355	0.332	13
Rule-blind − permuted, median [IQR]	0.120 [0.106, 0.131]	0.128 [0.115, 0.142]	—
test-set bootstrap 95% CI (GBT)	[−0.078,0.199] (includes zero)		—

*Note.* Single home; leakage-free labels (thresholds fit on the training segment only); one fixed temporal 70/30 split; 20 random seeds; $n_{\mathrm{test}}=504$ episodes; full-sample class prevalence 0.766/0.205/0.029. Point estimates are medians over seeds; the rule-blind−permuted gap carries its seed IQR and a test-set bootstrap 95% CI. Removing the four threshold-defining features (Table 18) collapses macro-F1 toward the permuted floor for both model families.

**Table 18:** Features removed in the rule-blind configuration (Table 17).

Feature	Unit	Raw/Derived	Role in Label Rule (Threshold)	Why Excluded from Rule-Blind
house-total mean	kW	derived (episode mean)	EnergyAnomaly if >μ+2σ of the training house-mean	the variable the energy-spike threshold is applied to
house-total maximum	kW	derived (episode max)	co-defines the EnergyAnomaly spike	monotone in the same house-power signal
wine-cellar mean	kW	derived (episode mean)	StandbyPhantomLoad if in $\left(5\;W,q_{15}\right]$ idle band	the variable the standby band is applied to
home-office mean	kW	derived (episode mean)	StandbyPhantomLoad if in $\left(5\;W,q_{15}\right]$ idle band	the variable the standby band is applied to

*Note.* Each removed feature is a direct input to the deterministic label threshold, so a classifier given them recovers the labelling rule rather than diagnosing a fault. Full labelling pseudocode is in Appendix C.

### 5.5. Labeled-Fault Validation on LBNL FDD (Fan Coil Unit, Simulation Ground Truth)

The CASAS results rest on proxy labels and the HomeC results on physics-grounded labels; neither provides expert ground-truth fault annotations. To close that gap, we evaluate on the LBNL Fault Detection and Diagnostics dataset [49], the largest public collection of labeled HVAC fault time series, using its Fan Coil Unit (FCU) subset: 29 monitored points sampled at one-minute resolution over a full year, with ground-truth labels for one fault-free baseline and 48 faulted scenarios. The dataset ships with a Brick model of the FCU, so its points map onto our ontology vocabulary, and its command points (valve, damper, and setpoint commands) instantiate the intervention space of Section 3.6. From the 48 scenarios, we use fifteen files (a fault-free baseline and fourteen faulted scenarios) grouped into seven classes (fault-free, sensor bias, valve stuck, valve leak, damper stuck, coil fouling, and airflow restriction), augment the 29 points with seven ontology-derived features (setpoint deviations and command-versus-position mismatches), downsample to 30-min resolution, and use a temporal 70/30 split *within each scenario*; the held-out test set contains 78,840 samples. This split shares a scenario between train and test, so it tests within-scenario temporal generalization, not held-out-scenario or held-out-severity generalization; we therefore additionally evaluate held-out-scenario and held-out-severity protocols below; block-bootstrap confidence intervals on the F1 and ECE gaps are reported below.

**Figure 4:**
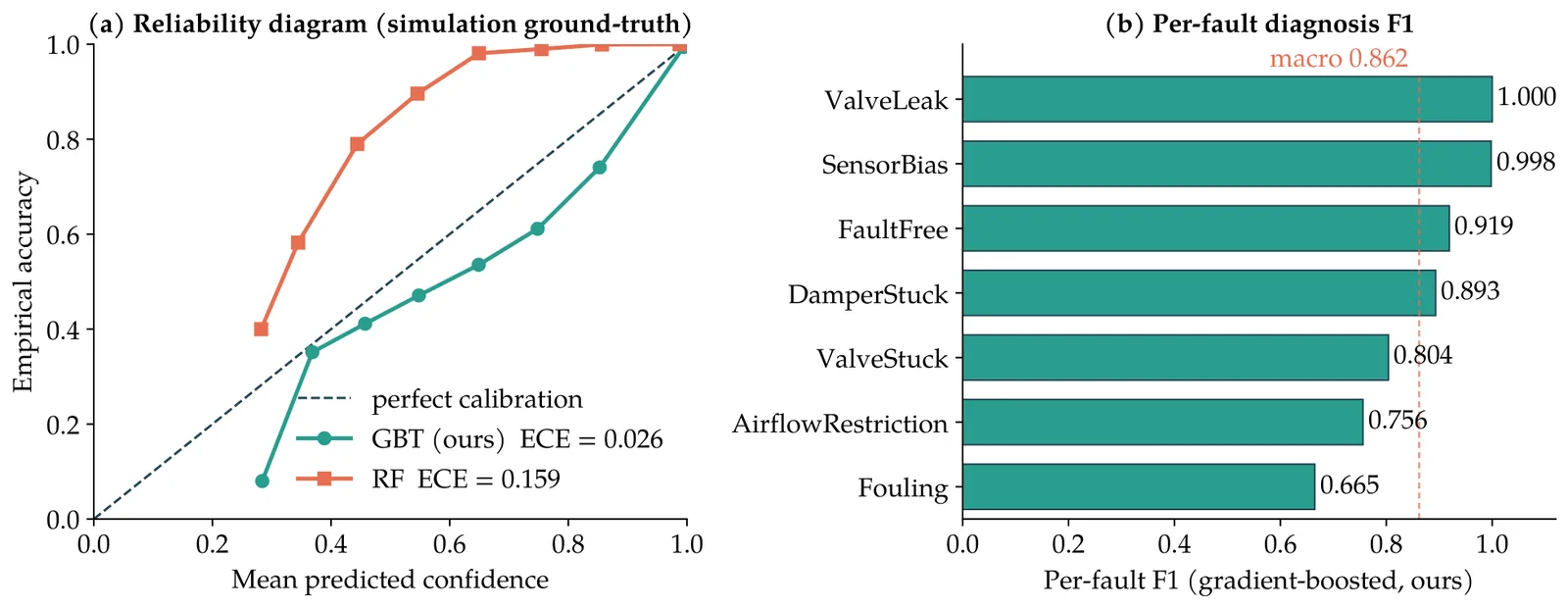
LBNL FCU fault-classification calibration. *Note.* (**a**) Reliability diagram: GBT (ours) tracks the diagonal (ECE 0.026) while the comparably accurate random forest does not (ECE 0.159). (**b**) Per-fault F1 for GBT (macro 0.862).

*The calibration contribution holds on labeled faults, with statistically resolved gaps:* Table 19 reports the result from a clean rerun of the released 30-min pipeline with the exact documented model configurations. GBT reaches macro-F1 0.862 [0.850, 0.873] and a tuned random forest 0.846 [0.834, 0.858]; the F1 gap is small but resolved (+0.016 [0.009, 0.023]). The decisive difference is calibration: GBT attains an expected calibration error (ECE) of 0.026 [0.019, 0.032] against the random forest’s 0.159 [0.148, 0.170] at comparable accuracy, approximately six-fold lower, with the RF−GBT ECE gap 0.134 [0.119, 0.148] excluding zero (Figure 4). On ground-truth-labeled faults, therefore, the GBT classifier delivers fault probabilities a building operator could act on, which a comparably accurate but poorly calibrated random forest does not. Per class, the sensor-bias fault (the building-systems analogue of SensorDegradation) is recovered almost perfectly (F1 0.998), and the actuator faults, whose corrective interventions are the ontology command points, are diagnosed strongly (valve leak 1.000, damper stuck 0.893, valve stuck 0.804); the physically subtle coil-fouling fault is hardest (0.665). The ECE uses 15 equal-width bins on the top-label confidence, without post hoc probability calibration and with empty bins skipped; all confidence intervals are block-bootstrap (2.5/97.5 percentiles, 500 resamples, seed 42) over scenario–day units rather than individual timestamps, so temporal autocorrelation does not narrow them artificially. *Provenance:* an earlier version reported ECE 0.019 (GBT) and 0.183 (RF), obtained by decimating to 30-min resolution (keeping every thirtieth one-minute sample); the released pipeline instead block-averages each 30-min window and, with the documented model configurations, produces 0.026 (GBT) and 0.159 (RF). We report the released-pipeline values throughout, and the block-bootstrap intervals above are computed from those same predictions (src/lbnl_experiment.py, scenario–day resampling, 500 draws, seed 42). These labels are ground-truth by construction but simulation-derived (HVACSIM+), not field-measured, as the scope note below makes explicit.

*Scope:* the FCU data are generated by HVACSIM+ simulation, so the labels are ground-truth by construction but not field-measured. Equally important, the ECE reported here measures the calibration of a *multiclass fault classifier’s* probabilities; it is consistent with, but does not by itself validate, the *counterfactual intervention* probability of Section 3.6, which is tested only on the synthetic SCM. The LBNL collection’s roof-top-unit subset also provides field measurements; cross-system transfer (FCU → air-handling/boiler plant) [50] and the appliance datasets of Section 6.4 are the immediate next steps.

**Table 19:** Labeled-fault diagnosis and calibration on the LBNL FDD Fan Coil Unit.

Method	Macro F1	Accuracy	Brier	ECE
GBT (ours)	**0.862**	**0.875**	**0.161**	**0.026**
Random forest	0.846	0.847	0.274	0.159
Logistic regression	0.548	0.539	0.502	0.043

*Note.* Seven fault classes; leakage-controlled temporal 70/30 split; 30-min resolution; $n_{\mathrm{test}}=$ 78,840; HVACSIM+ simulation-derived ground-truth labels, not field-measured. Values are from a clean rerun of the released pipeline. Block-bootstrap 95% CIs (scenario–day units) accompany the F1 and ECE gaps in the text; GBT’s ECE is approximately six-fold lower than the random forest’s.

*Generalization beyond the scenario-matched split:* the result above uses a temporal 70/30 split *within* each simulation scenario, so a reviewer may reasonably ask whether scenario-specific operating trajectories persist across train and test. We therefore re-evaluate under two stricter protocols on the same seven-class data (src/lbnl_generalization.py; matched balanced train and test of 5000 episodes per class, GBT and RF as above). Under a **held-out-scenario** protocol—entire simulation files are assigned to either training or test, so no scenario is split—GBT macro-F1 falls from 0.840 to 0.332 and its calibration collapses (ECE 0.029→0.372); RF degrades similarly in accuracy (macro-F1 0.828→0.348) while its ECE stays near 0.15. Under a **held-out-severity** protocol—the sensor-bias class is trained only on the ±2 °C files and tested on the unseen ±4 °C files—performance and calibration are essentially unchanged (GBT macro-F1 0.841, ECE 0.026), so the estimator extrapolates across the *severity* of a known fault but not across an *unseen scenario*. This is an honest and important limitation: the well-calibrated LBNL result (ECE 0.026) holds under the scenario-matched split reported above and should not be read as evidence of calibrated generalization to unseen operating scenarios. The held-out-scenario protocol is also stringent by construction, because for some classes the held-out file is a different fault polarity (e.g., stuck-open vs. stuck-closed valve), so it bounds the difficulty rather than isolating a single factor. Table 20 reports all three protocols.

### 5.6. Command-Point Counterfactuals Under Feedback Control (LBNL FCU)

We evaluate the counterfactual layer directly on the cooling-valve command point of the LBNL FCU dataset. Because the valve command is produced by a feedback controller rather than randomly assigned, we interpret the estimates as *observational command-point counterfactuals under feedback control*, not as identified causal effects. A GBT response model is trained on fault-free periods to predict the *h*-step change in room temperature from the observed thermal state and lagged temperatures (h∈{5,15,30,60} min; temporal 60/20/20 split; day-block bootstrap confidence intervals). A circularity check precedes the analysis: in the stuck-valve files, the valve command keeps varying while the valve position is externally fixed, so the command does not define the fault label and can serve as an intervention variable (the command-versus-position mismatch, which does encode the fault, is excluded from the counterfactual query).

*Factual reconstruction:* on held-out fault-free periods, the full response model improves on a persistence baseline at every horizon with separated confidence intervals (Table 21). Removing the valve-command feature leaves factual accuracy essentially unchanged, indicating that short-horizon temperature is dominated by the observed thermal state and that valve-command variation is limited during normal operation.

*Direction of the observational intervention effect:* increasing the cooling-valve command produces the physically expected temperature decrease for 88.5% [85.3, 91.5] of cases at the 30-min horizon, but the estimated sign is frequently reversed at 5 and 15 min. This is the signature of feedback confounding: the controller opens the valve in response to elevated room temperature, creating a short-horizon association opposite to the eventual cooling response.

*Controlled fault-injection stress test with externally imposed valve positions:* we complement the observational analysis with the stuck-valve fault files. Every fault file shares the same simulated weather and setpoint schedule: outside-air temperature and cooling setpoint are identical across files to numerical precision, so operating conditions are matched and the response can be attributed to the imposed valve position within the simulation. The stuck-closed condition yields a substantially higher mean room temperature (77.8 °F) than the valve fixed at 20% or above (≈70 °F), a monotone dose–response. Queried under do(valve=k) on matched fault-free states, the counterfactual model reproduces this direction: its predicted response is monotone in *k* and cooler for larger *k*, with a statistically resolved effect (do(0)−do(0.5)=+0.15 °F [0.14, 0.16] at 30 min and +0.27 °F [0.26, 0.28] at 60 min, day-block CIs). The recovered magnitude is a small fraction of the 8.2 °F steady-state gap, because the 30–60 min horizon captures only the initial response and the fault-free training data rarely exercise the valve; and the healthy model extrapolates poorly to the extreme stuck-closed regime (predicted–observed correlation 0.03 on the overheating file). A placebo that shuffles the valve across time produces no consistent effect.

*Interpretation:* the model reconstructs factual FCU dynamics and recovers the expected cooling direction at operationally meaningful horizons, while transparently exposing the limits of counterfactual estimation from feedback-controlled observational data: short-horizon confounding and distribution shift toward the fault regime prevent recovery of the full intervention magnitude. We report this as honestly bounded real-data support for the command-point intervention framing, not as an identified causal effect; damper- and setpoint-command replications are the natural next robustness steps.

**Table 21:** Command-point counterfactual on the LBNL FCU cooling valve.

Model	5 min	15 min	30 min	60 min	Sign-Consistency (30 min)
Persistence	0.094	0.221	0.399	0.692	—
Command-removed	0.046	0.139	0.276	0.544	—
Full factual model	**0.045**	**0.136**	**0.275**	0.546	—
Counterfactual (valve ↑)	—	—	—	—	**88.5%** [85.3, 91.5]
Placebo (shuffled valve)	—	—	—	—	no consistent effect

*Note.* Fault-free training; temporal 60/20/20 split; MAE of the *h*-step room-temperature change (°F) on held-out fault-free periods; day-block bootstrap 95% CIs. The full model beats persistence at every horizon; the valve-command feature adds little to factual accuracy. The counterfactual sign is physically correct at ≥30 min but reversed at short horizons (feedback confounding).

*Secondary robustness: a negative-control replication on the outside-air damper:* we repeated the command-point evaluation for the outside-air damper (command FCU_DMPR_DM, position FCU_DMPR) with outside-air flow (FCU_OA_CFM) as the primary response (Table 22). The controlled stuck-damper experiments show a monotone operational response in mean airflow (imposed damper 0%→100%: 0.9→680 CFM), but this is concentrated in the upper tail: the median airflow stays near 1 CFM at every severity because outside air flows only when the fan is active, and the response jumps sharply at 100% (monotone, not smooth). The counterfactual model trained on fault-free operation does not recover this relationship: the estimated airflow effect of a damper-command intervention is ≈0 CFM (median 0.000, day-block 95% CI [0.000,0.000], P(Δ>0)=0%), and the full model beats persistence only at the shortest horizon. The cause is a *positivity (support) violation*, not the absence of a physical effect: in fault-free operation the damper never opens beyond 30% (position median 0.000, 99th percentile 0.300), so the 50–100% stress levels lie entirely outside the training support. A mechanistic check confirms this: the model fails to recover even the command→position response (P(position↑)=0% under a +0.2 command intervention), so the failure is a lack of observational variation, not an actuator-to-airflow mapping error. In contrast to the cooling valve, whose command varies enough in normal operation to recover the expected direction at longer horizons, the damper is a *negative control*: it bounds the cooling-valve result by showing that a physically real intervention effect cannot be recovered when the relevant command is dormant in the training data. This is a support limitation of observational command-point counterfactuals under feedback control, not evidence that the damper has no effect.

## 6. Discussion

We organize the discussion around three questions: what the benchmark-validity audit reveals, what the calibration experiments demonstrate, and what remains unverified by the experiments. The first two are treated here and in Section 6.1; the third is consolidated in Section 6.3.

The benchmark-validity audit (Section 5.1) clarifies what CASAS can and cannot establish. Under a shared feature set, the model family is immaterial (0.36–0.38 rule-blind, 0.92–0.97 rule-exposed), and the high scores are carried by features that re-encode the proxy rules. The benchmark therefore measures semantic-transfer-under-leakage, not fault-detection accuracy, and we report it as such. This does not make the ontology unimportant; it operates where benchmark accuracy cannot register it, as Section 6.1 sets out.

We make no architecture-advantage claim: under a shared feature set the scores coincide (Table 15), so the decisive signal on CASAS is the proxy-rule encoding, a property of the benchmark rather than the model, and this is why the framework’s evidentiary weight rests on the calibration results.

Those calibration results answer the second question. The counterfactual layer returns a calibrated probability rather than a label or ranking, which is the framework’s main departure from prior smart-home work. On the controlled SCM, it reaches a Brier skill score of 0.369 and, head-to-head against an isotonic-calibrated GBT baseline [38,39], remains the better-calibrated forecaster (Brier 0.158 vs. 0.204), so the advantage is a genuine comparison rather than an absent-competitor artifact. On the LBNL FCU benchmark, a *different* estimator—a GBT multiclass fault classifier, not the factor-graph intervention model—is well-calibrated under a scenario-matched split but not under a held-out-scenario split (Section 5.5); these are two distinct calibration results, not one joint claim.

The third question—what remains unverified—is taken up in the subsections below (the boundary of the ontology’s contribution, the cross-dataset transfer gap) and consolidated in Section 6.3; in short, the CASAS labels are proxy injections, so those results characterize semantic transfer, not anomaly prevalence, and the intervention-success calibration claim does not extend beyond the controlled SCM, while the separate multiclass fault-probability calibration result is supported only on the simulation-derived LBNL benchmark.

### 6.1. Where the Ontology’s Contribution Actually Lies

A natural question is whether the ontology contributes anything beyond the proxy-rule signal that the audit isolates. On CASAS, the benchmark cannot separate the two, which is why we argue the ontology’s value from the counterfactual layer rather than from benchmark accuracy. Its contribution lies in three places that benchmark accuracy does not measure.

1. **Shared vocabulary for transfer.** Semantic normalization (the step that carries most of the gain) is ontology-mediated: the mapping from home-specific IDs to ontology types is the precondition for any cross-home transfer, so attributing the gain to “semantic features” rather than “ontology” is presentation, not mechanism.
2. **Causal-graph structure.** The graph (Section 3.4) is read off ontology-typed nodes and the four relation types; without it, edges would be ad hoc or learned rather than auditable. This serves interpretability and intervention reasoning, not raw accuracy.
3. **Counterfactual layer.** The factor graph (Section 3.6) draws interventions from ontology prevents edges and reasons over ontology-typed variables; without it, the intervention space is unprincipled. The ontology’s value becomes measurable in the Brier-calibrated SCM evaluation (Section 5.3).

Had the goal been to maximize benchmark F1, semantic features without the ontology-constrained causal and counterfactual layers would have sufficed; the ontology exists to enable the calibrated counterfactual layer that no baseline classifier provides.

*A measured demonstration:* the executable OWL/SHACL prototype released with the paper makes this contribution measurable under controlled fault injection (details in Appendix A). The ontology (v0.3.0; 32 classes, 13 object properties, 41 individuals) is OWL-RL consistent with no unsatisfiable classes and zero SHACL violations, and it answers 14/14 competency questions; for example, the SPARQL query *“which interventions prevent CoolingLoss?”* returns exactly {CloseWindow, RaiseSetpoint} through the prevents relation. In a leave-one-home-out study over five heterogeneous homes with unseen raw sensor identifiers, semantic abstraction is necessary (a raw-ID classifier collapses to macro-F1 0.065), and strong tabular baselines (logistic regression, random forest) match the ontology on classification (macro-F1 1.0). The ontology’s distinctive, measurable value lies elsewhere: only the ontology-guided engine also selects the correct cause and the correct *cause–intervention pair* (both 1.0); the flat classifiers do not emit a cause node at all, so these outputs are not produced rather than scored zero (Table A1). This is because its admissibility constraints (targetsCause, requiresEvidence) tie each candidate intervention to the diagnosed cause, something a flat class label cannot do. This is demonstrated only under controlled fault injection, not on real streams; a relation-shuffle permutation test gives p=0.083 over 24 permutations, so the effect is indicative rather than conclusive at the five-home scale. *Where the ontology constraint is and is not decisive:* an ablation on the controlled SCM (Section 5.3), under identical splits and seeds, compares three variants: the ontology-guided selector (apply the intervention the ontology marks admissible for the diagnosed cause), an unconstrained factor graph (pick the intervention with the highest P(normal∣do) regardless of cause), and a flat calibrated classifier. On this clean SCM, the first two are identical (CF-Success 0.770, Brier 0.158, 0% inadmissible interventions): the outcome-optimal intervention already coincides with the cause-admissible one, so the explicit ontology constraint is redundant here. The flat classifier, lacking the structured graph, selects an intervention that does not target the diagnosed cause 20.1% of the time and attains lower CF-Success (0.685). The ontology’s benefit is therefore realized through the structured causal graph, which drives the inadmissible-intervention rate from 20.1% to 0%, rather than through accuracy or calibration gains on the clean SCM; the explicit relation constraints become decisive only under distribution shift, where the cross-home study shows the relation-free variant loses the cause–intervention pairing entirely (Table A1).

### 6.2. The Behavioral-Context Gap: Cross-Dataset Transfer to Aruba

Aruba is an activity-recognition dataset, not a fault-diagnosis one; we use it only to probe the *semantic-vocabulary-transfer* claim in isolation—whether an ontology-aligned model carries over to a home with a different behavioural distribution when the label semantics are held fixed. Zero-shot transfer to Aruba returns macro-F1 near zero, which shows that vocabulary alignment is necessary but not sufficient: sensor *types* map through the ontology, but the temporal-statistical distribution of which types fire does not transfer (in Aruba the night-window outside-door rate is effectively zero, versus 0.558 in hh101–hh110). A controlled domain-adaptation study—full protocol, label-space audit, and per-class results in Appendix D—refines this into three honest findings: unsupervised CORAL alignment recovers part of the covariate shift (macro-F1 0.290→0.383, 95% CI on the gain [0.059,0.127]); a mere 5% of labelled target data dominates all source knowledge (0.574, against a 0.641 supervised upper bound); and naive source–target pooling causes statistically supported *negative transfer* (−0.093, CI [−0.117,−0.048]), which a pretrain-then-fine-tune scheme avoids (+0.003, CI [−0.084,0.067]). This is a diagnostic probe of the transfer mechanism, not a fault-detection result; behavioural-dynamics alignment on a small target sample is the principal direction for follow-up work [51].

### 6.3. Threats to Validity

This work uses a deterministic proxy anomaly construction (Section 4.3) because expert-annotated faults are unavailable for CASAS hh101–hh110. The proxy rules are explicit and verifiable, but they carry rule-like temporal signatures that the classifier may learn directly: AbnormalDoorEvent is anchored to door-open events in the 02:00–03:59 window, SensorDegradation to motion-event sequences with roughly two-second inter-event gaps, and OccupancyModeConflict to motion events during Leave_Home or Enter_Home spans. Because these signatures are deterministic, the high F1 obtainable on this benchmark (the audit of Section 5.1) should be read as performance on a controlled semantic-transfer benchmark, not as evidence of operational fault-diagnosis accuracy. A classifier that learns “a door event between 02:00 and 04:00 implies AbnormalDoorEvent” is partly recovering the injection rule rather than discovering an underlying fault mechanism. We accept this limitation and frame the contribution as a methodological probe: under what conditions does cross-home semantic-transfer work, and what does calibrated counterfactual reasoning add on top of classification? We also note that semantic abstraction hides raw device identifiers but is not, on its own, a privacy guarantee: a formal threat model (linkage and membership-inference attacks against behavioral features [52], and the trust boundary between edge normalization and downstream inference) remains future work, and we make no formal privacy claim here.

Three properties keep the proxy benchmark informative despite this limitation. First, the rules are disclosed, so the gap between rule-learning and structural diagnosis is auditable. Second, the rules are applied uniformly across the ten homes, so cross-home transfer must still bridge the heterogeneity of sensor placement, behavioral rhythm, and instrumentation density, the effect the framework is designed to probe. Third, the counterfactual evaluation (Section 5.3) uses a separate controlled SCM in which ground-truth interventions are known by construction, so the calibration claims rest on that controlled experiment rather than on the CASAS classification numbers. A genuine deployment evaluation would require expert-annotated faults on a longitudinal dataset with diverse fault types, ideally with paired interventional outcomes; we make no such claim here. Finally, expert validation of counterfactual quality (plausibility, actionability, trustworthiness) has not yet been conducted; we identify it as required future work rather than claiming any preliminary result.

The empirical evidence in this paper validates two things: a semantically normalized tabular classifier (Section 5.1, Section 5.2, Section 5.4 and Section 5.5) and probability calibration on a synthetic SCM and on simulated HVAC faults. It does not yet validate the full “ontology-guided” pipeline end to end. We release an executable OWL/SHACL ontology prototype, with reasoner-consistency checks and competency questions, together with the reproducible controlled-SCM counterfactual layer (Section 5.3), but both are evaluated only under controlled fault injection; we do not yet report mapping-coverage or inter-annotator analysis [53], nor a reproducible run of the causal graph and belief-propagation convergence on real streams (LBP convergence is characterized on representative graphs in Appendix B). Until the ontology and causal graph are evaluated end to end on real streams, they should be read as a specified architecture validated only under controlled conditions rather than a fully demonstrated system. Accordingly, the title scopes this paper to its validated core, a reproducible benchmark-validity audit and calibration study, while the ontology-guided framing is presented as a design validated only under controlled conditions; full end-to-end ontology evaluation on real streams remains future work.

### 6.4. Roadmap to Deployment: Three Open Validations

Section 5.5 supplies a simulation-derived labeled-fault test (HVACSIM+ FCU), which supports the multiclass-classifier calibration claim but does not by itself validate the counterfactual module (Section 5.5). We close the discussion by naming the validations that still remain.

The real-data evaluation here covers a single home (HomeC, Section 5.4); a single site cannot exercise the leave-one-home-out protocol that the cross-home claim ultimately requires. The natural next benchmark is appliance-level sub-metered data from REDD [44] and AMPds2 [45], on which physics-grounded anomaly rules can be applied uniformly across homes, appliance-left-on thresholds informed by energy-efficiency standards (e.g., ASHRAE 90.1), refrigerator-door-open per DOE 10 CFR 430, phantom load per the LBNL standby report, and degradation per NREL building-performance studies. This is the experiment that would confirm or refute that the *proxy-label circularity* found on HomeC (Section 5.4) recurs across independent homes, and that the ontology transfers across genuinely independent deployments.

The calibration and selection results (Section 5.3, Figure 3) are obtained on a controlled SCM because, in real homes, the outcome of an intervention cannot be observed without performing it. Closing this gap requires interventional ground truth: an instrumented actuation study in which candidate actions (closing a window, adjusting a setpoint, invalidating a sensor) are actually executed and their effects logged, or the use of operator-action logs paired with subsequent outcomes. Until such data exist, we scope every calibration claim to the synthetic SCM and make no quantitative claim about real-world CF-Success.

Diagnosis costs O(T|E|) per episode (Section 3.5), which is promising for edge deployment [54,55], but we report no empirical operating figures. A deployment study should measure inference throughput (episodes per second), peak memory, and concurrent multi-home capacity on representative edge hardware (e.g., Raspberry Pi- or Jetson-class devices), since the Special Issue’s intelligent-sensor setting makes these as consequential as accuracy. We regard this profiling as a prerequisite for any real-time claim.

These three validations are independent and additive: (1) tests cross-home generalization on real measurements, (2) tests the counterfactual layer against real interventional outcomes, and (3) tests whether the pipeline meets edge-IoT operating constraints. Keeping them out of the present results (rather than substituting synthetic proxies) is a deliberate choice so that demonstrated capability and future work stay clearly separated.

## 7. Conclusions

We presented a framework that combines ontology-grounded semantic discretization, an ontology-constrained causal graph with belief-propagation inference, and a factor-graph counterfactual module with Brier calibration, aimed at the cross-home anomaly-diagnosis problem in smart-home sensor streams.

The contributions are threefold. First, a reproducible benchmark-validity audit of cross-home smart-home fault diagnosis, with disclosed proxy rules, leave-one-rule-out and permuted-label controls, and a shared-feature comparison across model families, which shows that high CASAS scores are largely proxy-rule leakage rather than transferable diagnosis. Second, a calibrated counterfactual intervention layer, validated on a controlled structural causal model (intervention-success Brier skill score 0.369); and, as a separate result, a well-calibrated GBT *multiclass fault classifier* on simulation-derived LBNL FDD faults (approximately six-fold lower calibration error, ECE 0.026 vs. 0.159, at comparable accuracy). These are two distinct calibration results—an intervention-success probability on the SCM and a multiclass fault probability on LBNL—not a single joint claim; the LBNL command-point analysis (Section 5.6) is a third and separate result, an observational response model under feedback control rather than an identified causal effect. Third, a fully reproducible release: documented preprocessing, the audit pipeline, a controlled-SCM counterfactual emulator, and an executable ontology prototype, so that every reported number regenerates from public data. Supporting these, we introduce an ontology-grounded framework (a Brick/SAREF-extended vocabulary, semantic discretization, and an ontology-constrained causal graph) that we specify and validate only under controlled conditions.

On CASAS hh101–hh110 a benchmark-validity audit shows that the high cross-home F1 obtainable under the disclosed proxy rules is largely leakage: removing the rule-encoding features collapses macro F1 from 0.936 to 0.383, only 0.120 above the permuted-label floor, and three model families coincide under a shared feature set. We therefore report no headline accuracy for this benchmark and treat it as a semantic-transfer and validity probe. On real appliance-level data (HomeC, UMass Smart*) with physics-derived threshold labels, the same audit recurs: under leakage-free labels a rule-exposed macro-F1 of 0.963 collapses to 0.445 when the four label-defining features are removed, against a permuted floor of 0.355 (the rule-blind−permuted gap is not distinguishable from zero), a second instance of proxy-label circularity rather than independent validation. The distinguishing contribution is the calibrated counterfactual layer, which on the controlled SCM attains a Brier skill score of 0.369 for intervention-success probabilities. Separately, on the LBNL FDD Fan Coil Unit (HVACSIM+ simulation-derived ground-truth labels, not field-measured) a conventional GBT multiclass fault classifier attains an ECE of 0.026 [0.019, 0.032] against a comparably accurate random forest’s 0.159 [0.148, 0.170] (an approximately six-fold reduction; RF−GBT ECE gap 0.134 [0.119, 0.148], excluding zero)—a distinct classifier-calibration result, not a second validation of the counterfactual layer. The counterfactual recommendations themselves are validated only under controlled or simulated conditions, not in deployed homes. On the same FCU we also evaluate the counterfactual layer directly on the cooling-valve command point (Section 5.6): the response model beats persistence and recovers the physically correct cooling direction at ≥30 min, while feedback confounding and distribution shift transparently bound what observational command-point counterfactuals can claim.

The proxy benchmark, the synthetic counterfactual evaluation, and the absence of expert-annotated faults confine the conclusions to methodological claims. Future work targets (a) expert-annotated fault evaluation on longitudinal smart-home datasets such as REDD [44] or AMPds2 [45]; (b) domain-adaptive calibration for behavioral-context shift, addressing the Aruba-type transfer gap; (c) physical-actuation experiments that validate counterfactual interventions against real ground truth; and (d) a real-time deployment study under operational constraints. We position the framework as a reproducible methodological substrate on which these follow-up evaluations can be built. Three groups should find the study directly useful. Researchers working on sensor-based fault diagnosis gain a reusable audit protocol for checking whether a benchmark measures fault detection or label-rule recovery before results are published. Dataset curators gain concrete evidence that proxy-annotation rules leak into the feature space, and a template for disclosing those rules. Practitioners gain an evaluation procedure for deciding whether the probabilities are sufficiently calibrated for a specified operating condition, together with an explicit statement of the conditions under which the present probabilities were validated; the results here do not establish deployment readiness.

## Figures and Tables

**Table 1:** Symbols and notation.

Symbol	Meaning
$X_{h}$	Raw event stream for home *h*
$\varphi_{h}$	Home-specific semantic mapping function
*S*	Shared semantic state sequence
$W_{j}$	Diagnosis window for episode *j*
*k*	Window length in events
*s*	Window stride (step) in events
*C*	Closed-set candidate cause space
$f_{j}\in R^{d}$	Episode feature vector (*d* varies by dataset)
$y_{j}$	Structured diagnosis tuple $\left(a_{j},c_{j},q_{j},r_{j}\right)$
G=(V,E)	Ontology-constrained causal graph
*e*	Observed semantic evidence

**Table 4:** Semantic variables and discretization rules.

Variable	Raw Source	States/Bin Boundaries	Calibration
Inter-event gap Δt	Timestamps	eight bins: 0–10 s, 10–30 s, 30 s–1 min, 1–5 min, 5–30 min, 30 min–2 h, 2–8 h, >8 h	Home-adjusted Δt statistics
Time of day	Timestamps	night 0–5 h, day 6–17 h, evening 18–23 h	Fixed boundaries
Event density	Windowed counts	normal, elevated, burst	Per 15-event window
Occupancy evidence	PIR/motion	none, transient, sustained	Event persistence
Door activity	Contact sensor	open, closed, transition	Direct mapping
Open duration (window/door)	Contact sensor	Brief <1 min, Sustained 1–10 min, Extended >10 min	Fixed symbolic thresholds
Setpoint deviation ΔT	Room temp. − setpoint	within band, below band, above band	τ=μ±1.5σ, per-home baseline period
Appliance evidence	Item events	inactive, expected, inconsistent	Co-occurrence

**Table 5:** Proposed inference configuration for the full factor-graph pipeline.

Component	Choice	Rationale
Graph form	Discrete factor graph	Tractable, interpretable
Structural relations	CPTs over semantic states	Aligns with the ontology
Algorithm	Loopy belief propagation	Efficient on sparse graphs
Graph scope	Local anomaly subgraph	Focused context
Fallback	Reduced local inference	Handles weak convergence
Prior (*U*)	Bounded uniform	Conservative default
Parameter estimation	MLE from seen homes	Deployment-realistic

*Note.* This table specifies the *proposed* full configuration. The conditional probability tables, MLE parameter estimation, loopy belief propagation, and fallback strategy are part of the intended end-to-end system and are *not* executed on the reported dataset experiments, which evaluate the components separately (the CASAS and HomeC audits use feature-fed classifiers; the calibrated counterfactual results come from the controlled SCM emulator of Section 5.3 and the ontology prototype of Appendix A). Fitting these tables on real streams is future work.

**Table 6:** Components of the five evaluation pipelines.

Pipeline	Data & Label Source	Unit & Features	Train/Test Split	Model(s)
CASAS audit	CASAS hh101–hh110; disclosed proxy-rule labels	50-event episodes; 16 count features (+3 rule features)	Leave-one-home-out	GBT, RF, LR
HomeC audit	UMass Smart* HomeC; physics-threshold labels	5-min episodes; 13 power features	Temporal 70/30, 20 seeds	GBT, XGBoost
LBNL FCU	LBNL FDD (HVACSIM+ simulation); simulated ground truth	1-min rows; 36 sensor/derived features	Within-, held-out-scenario, held-out-severity	GBT, RF, LR
SCM emulator	Synthetic SCM; known do-interventions	30,000 episodes; 4 evidence dimensions	70/30 on episodes	Factor graph, calibrated GBT
Ontology prototype	Synthetic fault bank; 5 homes	Ontology individuals; controlled fault injection	Leave-one-home-out	Ontology engine, ML baselines

**Table 7:** What each evaluation does and does not establish.

Pipeline	Ground Truth	What It Establishes	What It Does Not Establish
CASAS audit	Proxy rules	Benchmark leakage; that model family is not decisive under a shared feature set	Real fault detection; expert-verified cross-home transfer
HomeC audit	Physics thresholds	The same proxy-label circularity recurs on real appliance measurements	Real fault detection; independent validation
LBNL FCU	Simulation	Multiclass fault calibration under the scenario-matched split (GBT ECE 0.026)	Calibrated generalization to unseen scenarios (degrades sharply); field faults
SCM emulator	Synthetic (known interventions)	Calibrated intervention-success probability (Brier skill 0.369)	Real-world intervention outcomes
Ontology prototype	Synthetic fault bank	Correct cause and cause–intervention selection under controlled injection	Real-stream, end-to-end diagnosis

*Note.* No pipeline rests on expert-verified real-world faults; the intervention-success calibration claim is supported only by the controlled SCM, while the LBNL simulation supports the separate multiclass fault-probability calibration result.

**Table 8:** Dataset and home-selection summary.

Item	Value
Dataset	CASAS smart-home dataset (hh101–hh110)
Protocol	Leave-one-home-out (10 homes)
Event representation	Shared semantic event space
Episode unit	50-event non-overlapping episodes (audit, Section 5.1)
Evaluated classes	SensorDegradation, OccupancyModeConflict (AbnormalDoorEvent near-absent)
Primary sensors	Motion, door, item/appliance

**Table 9:** Proxy anomaly construction rules.

Class	Principle	Main Evidence
OccupancyModeConflict	Post-exit occupancy contradicts the away context	Door transition, persistence
AbnormalDoorEvent	Door activity inconsistent with the access pattern	Door transitions, timing
SensorDegradation	Flat, bursty, or inconsistent sensor behavior	Event bursts, repetition
ApplianceInconsistency	Appliance events inconsistent with activity	Item evidence, mismatch

**Table 10:** Baseline configurations for the shared-feature comparison.

Method	Model Family	Input Representation	Notes
GBT (ours)	GBT	Shared semantic feature set	Estimator used throughout
Random forest	Bagged trees	Shared semantic feature set	Strong tabular baseline
Logistic regression	Linear (standardized)	Shared semantic feature set	Linear reference

**Table 11:** Estimator hyperparameters by benchmark (random seed 42 throughout).

Benchmark	GBT (HistGradientBoosting)	Random Forest	Logistic Reg./XGBoost
CASAS (Table 12, Table 13, Table 14 and Table 15)	max_iter 200, learning_rate 0.1, max_depth 6	120 trees, class-weight balanced	LR: standardized, max_iter 1000, balanced
LBNL FDD (Table 19 and Table 20)	max_iter 150, learning_rate 0.12, max_depth 6	80 trees, max_depth 16	LR: standardized, max_iter 800
HomeC (Table 17)	max_depth 4	—	XGBoost: 100 trees, max_depth 4, learning_rate 0.1
SCM ablation (Section 6.1)	max_depth 4	—	—

*Note.* Balanced class weighting is applied only where noted; all other settings are library defaults. Logistic regression is always preceded by StandardScaler. Full definitions are in the released scripts under src/.

**Table 16:** Counterfactual intervention calibration on the controlled SCM (σ=0.4).

Method	CF-Success [95% CI]	Brier Score [95% CI]	Skill Score
Factor graph (do-calculus)	**0.770** [0.756, 0.782]	**0.158** [0.150, 0.165]	**0.369**
Gradient-boosted (indirect, labels)	0.685 [0.672, 0.700]	—	—
Gradient-boosted (isotonic-calibrated)	0.685 [0.672, 0.700]	0.204	0.183
Random	0.311	0.250	0.000

*Note.* The plain indirect classifier returns labels (Brier undefined); an isotonic-calibrated GBT baseline outputs a success probability for its chosen intervention, yet remains less well-calibrated than the factor graph (Brier 0.204 vs. 0.158).

**Table 20:** LBNL FCU generalization under three train/test protocols.

Protocol	Model	Macro F1	Accuracy	Brier	ECE
Within-scenario (70/30)	GBT	0.840	0.837	0.212	0.029
	RF	0.828	0.819	0.304	0.152
Held-out-scenario	GBT	0.332	0.415	0.916	0.372
	RF	0.348	0.410	0.797	0.142
Held-out-severity (2→4 °C)	GBT	0.841	0.837	0.211	0.026
	RF	0.782	0.776	0.300	0.117

*Note.* Balanced train and test of 5000 episodes per class, seed 42; GBT and RF configured as in Table 19. The within-scenario row reproduces the calibration finding of Table 19 under this matched-sample protocol.

**Table 22:** Outside-air damper negative-control replication (LBNL FCU).

Quantity	Value	Note
Fault-free damper position (median/p99/max)	0.00/0.30/0.30	never >30%
Stress severities out of support	50%,80%,100%	positivity violation
Observed airflow, do(damper0%→100%)	0.9→680 CFM (mean)	monotone, tail-driven
Model airflow effect (30 min)	≈0 [0.000, 0.000] CFM	P(Δ>0)=0%
Command→position recovered?	no (P↑=0%)	confirms support cause
Full vs. persistence (airflow)	better only at 5 min	no long-horizon gain

*Note.* The fault-free distribution never opens the damper beyond 30%, so the 50–100% stuck-damper stress levels are out of support and the observational counterfactual recovers no airflow effect despite a large (tail-driven) exogenous dose–response.

## Data Availability

The CASAS smart-home dataset used in this study is publicly available from the Center for Advanced Studies in Adaptive Systems at Washington State University, accessible upon registration at https://casas.wsu.edu/datasets/ (accessed on 6 July 2026). The released code currently covers the reproducible benchmark-validity audit (proxy-label generation, episode construction, leave-one-home-out evaluation, and the disclosed proxy rules of Section 4.3), the documented raw-to-feature preprocessing modules for all datasets (Section 4.2), the HomeC and LBNL FDD experiment scripts, an executable OWL/SHACL ontology prototype with a leave-one-home-out cross-home study under controlled fault injection, and the controlled-SCM counterfactual emulator that regenerates the calibration results of Section 5.3 under exact do-calculus inference with a fixed seed. A complete end-to-end deployment of the ontology reasoner and causal graph on real field streams remains future work. All reported audit numbers are regenerated by the released pipeline with a fixed random seed (42); the released episode table casas_windows.csv has SHA-256 checksum 0605637f0d939795fa2124ef16a18829ffacb0f5d7935ab4f3d0b994d1087241. The Python 3.10 environment (scikit-learn 1.7.2, XGBoost 3.2.0, NumPy 2.2.6, pandas 2.3.3, SciPy 1.15.3) is pinned in environment-lock.txt, and per-benchmark estimator hyperparameters are collected in Table 11; the exact revision behind every reported number is archived as release rev2-reviewer-response (commit 6bf18f2), so the results stay reproducible as the repository evolves. The command python check_all.py regenerates every source experiment and checks it against the values in this paper. The code and result files are available at https://github.com/saydirasulov-alt/calibrated-counterfactual-diagnosis (accessed on 6 July 2026) and are permanently archived on Zenodo at https://doi.org/10.5281/zenodo.21698507 (accessed on 6 July 2026).

## Abbreviations

   The following abbreviations are used in this manuscript:

ATE	Average Treatment Effect
BP	Belief Propagation
BSS	Brier Skill Score
CASAS	Center for Advanced Studies in Adaptive Systems
CF	Counterfactual
CI	Confidence Interval
DAG	Directed Acyclic Graph
ECE	Expected Calibration Error
F1	F-measure (harmonic mean of precision and recall)
FAR	False Alarm Rate
FCU	Fan Coil Unit
GBTs	Gradient-Boosted Trees
HAR	Human Activity Recognition
IoT	Internet of Things
LBNL FDD	Lawrence Berkeley National Laboratory Fault Detection and Diagnostics
LBP	Loopy Belief Propagation
LOO-CV	Leave-One-Home-Out Cross-Validation
LR	Logistic Regression
LSTM	Long Short-Term Memory
MAE	Mean Absolute Error
MAP	Maximum A Posteriori
MLE	Maximum Likelihood Estimation
RF	Random Forest
REDD	Reference Energy Disaggregation Dataset
SAREF	Smart Applications REFerence Ontology
SCM	Structural Causal Model
SHACL	Shapes Constraint Language
SSN/SOSA	Semantic Sensor Network/Sensor, Observation, Sample, and Actuator
XGBoost	eXtreme Gradient Boosting

## Appendix A. Executable Ontology Prototype: Reasoner and Competency Results

The released prototype (ontology_prototype/, run by src/run_all.py in ≈15 s) serializes a causal home ontology in OWL/TTL and validates it with an OWL-RL reasoner and SHACL shapes, then runs SPARQL competency questions and a leave-one-home-out (LOHO) cross-home diagnosis study under controlled fault injection. It checks objects (Home, Sensor, Device, Observation, Abnormality, Cause, Intervention) and relations (causes, enables, indicates, prevents, targetsCause, requiresEvidence, requiresContext, supportsCause), with Brick/SAREF/SOSA alignment by rdfs:subClassOf.

*Reasoner and shapes:* ontology v0.3.0: consistent = true; 32 classes, 13 object properties, 41 individuals; 0 unsatisfiable classes; 142 inferred subclass axioms; SHACL conforms with 0 violations. Competency questions: 14/14 passed (positive and negative queries).

*LOHO cross-home (five heterogeneous homes, unseen raw IDs, controlled fault injection; hierarchical bootstrap CIs).*

**Table A1:** Leave-one-home-out diagnosis on the controlled fault bank (210 episodes).

Variant	Macro-F1 [hCI]	Cause	Interv.	Pair
raw sensor IDs	0.065 [0.047, 0.083]	n/p	n/p	n/p
semantic, relation-free	0.733 [0.705, 0.753]	0.0	0.722	0.0
logistic regression	1.0 [1.0, 1.0]	n/p	0.722	n/p
random forest	1.0 [1.0, 1.0]	n/p	0.722	n/p
GBT	0.754 [0.723, 0.787]	n/p	0.722	n/p
ontology-guided	**1.0** [1.0, 1.0]	**1.0**	**1.0**	**1.0**

*Note.* n/p = not produced: a flat classifier emits only a class label and does not output a cause node, so its cause and cause–intervention-pair accuracies are undefined rather than zero (the intervention column is a fixed abnormality→action lookup, which the classifiers do produce). The *semantic, relation-free* row is an ablation of the ontology engine that does emit a cause but, with the relation constraints removed, recovers it at chance (0.0). The comparison the table supports is therefore narrow: on this controlled fault bank the structured engine recovers the cause–intervention pair, an output the flat baselines do not generate; it is not a claim that the ontology classifies better.

A relation-shuffle permutation test (24 permutations) yields cause-consistent coverage mean 0.361 and p=0.083 for the ontology-guided result, so the cross-home effect is indicative at this five-home scale rather than conclusive. *Limitation:* all ontology results are obtained under controlled fault injection on a synthetic fault bank; no claim is made about real-stream reasoning, whose end-to-end evaluation remains future work (belief-propagation convergence on representative graphs is characterized in Appendix B).

## Appendix B. Belief-Propagation Convergence Characterization

To characterize the inference step, we run loopy belief propagation (sum–product, binary variables, pairwise couplings) on synthetic factor graphs representative of the causal structure: an ontology-style layered topology (causes → abnormalities → observations with sparse cross-links that create loops) and, as a control, random Erdős–Rényi graphs of matched edge count, at 10, 30, and 60 variables. Each run logs the per-iteration maximum and mean message residual; convergence is declared when the maximum message change falls below $10^{-5}$ over an iteration, with a cap of 300 iterations and 6 random initializations per configuration. Damping uses $m^{\left(t\right)}=\left(1-\alpha \right)\;\hat{m}+\alpha \;m^{\left(t-1\right)}$. Table A2 reports convergence fraction and median iteration count for every configuration.

**Table A2:** LBP convergence on representative causal-structure graphs (6 initializations per cell).

Regime (30 Vars)	Metric	α=0	α=0.25	α=0.5	α=0.75
Moderate coupling	converged	100%	100%	100%	100%
	median iterations	56	**40**	52	96
Strong coupling	converged	0%	0%	0%	0%
	behaviour	osc.	osc.	osc.	osc.

*Note.* In the moderate-coupling regime matching the diagnostic potentials, LBP converges in every run across sizes and damping (fastest at α∈[0.25,0.5]); strong couplings oscillate at all damping levels. Across ontology-structured and random topologies at 10/30/60 variables, all 24 moderate-coupling configurations reach 100% convergence.

Two conclusions follow. First, in the operative (moderate-coupling) regime the inference is well-behaved: convergence is universal, the per-episode cost scales linearly with edge count as designed (O(T|E|)), and moderate damping (α∈[0.25,0.5]) minimizes iterations, supporting the default α=0.5. Second, LBP is not unconditionally convergent: under strong couplings, it oscillates regardless of damping, a well-known limitation that a deployed system must detect (e.g., via a residual monitor and a fallback). This characterizes the message-passing step on representative graphs; wiring the ontology-derived causal graph to real smart-home streams and repeating the study there remains future work.

## Appendix C. HomeC Physics-Threshold Labelling

The HomeC episode labels used in Section 5.4 are a deterministic rule over the five-minute episode aggregates, with all thresholds fit on the training segment only (the --leakage-free path). Class priority is EnergyAnomaly > StandbyPhantomLoad > Normal.

Because house_mean, house_max, wine_mean, and office_mean are simultaneously inputs to this rule and features supplied to the classifier (Table 18), a model can recover the rule directly; removing them is the rule-blind configuration of Table 17. The audit script src/homec_leakage_audit.py regenerates every HomeC number in this paper from HomeC.csv with the seeds listed above.

## Appendix D. Cross-Home Activity-Recognition Domain Adaptation (Aruba)

Source Tulum2, target Aruba; frozen temporal Aruba test set (last 30% of episodes); home-agnostic sensor-type episode features (motion and temperature event counts, mean/standard-deviation temperature, episode duration, unique-sensor count, event rate, hour-of-day, day-of-week). A RandomForest classifier is used for the majority, source-only, CORAL, target-only, pooled, and upper-bound rows; a fine-tunable log-loss linear model is used only for the pretrain→fine-tune control (so its absolute level is lower, and only the within-family gain is interpreted). The 5%-label methods report the mean over 12 episode-level samples with episode-bootstrap 95% CIs. Panel A is the strict five-class common space; Panel B adds the conservative harmonization Bed_Toilet_Transition → Bed_to_Toilet. Only Leave_Home is shared across all candidate source homes; multi-resident and meal-specific labels were not force-merged. Table A3 reports both panels.

**Table A3:** Cross-home domain adaptation, Tulum2→Aruba (macro-F1; frozen Aruba test).

Method (Target Labels)	Panel A (5-Class)	Panel B (6-Class)
Majority-prior (0%)	0.149	0.120
Source-only (0%)	0.290	0.381
CORAL alignment (0%)	0.383	0.381
Target-only (5%)	0.574	0.619
Pooled source +5%	0.478	0.508
Target upper bound (100%)	0.641	0.687
CORAL − source-only	+0.092 [0.059, 0.127]	−0.001 [−0.039, 0.04]
Pooled − target-only (5%)	−0.093 [−0.117, −0.048]	−0.111 [−0.144, −0.06]
Pretrain→fine-tune − target-only (within family)	+0.003 [−0.084, 0.067]	+0.056 [−0.025, 0.138]

*Note.* Naive source pooling causes negative transfer in both panels, whereas pretrain→fine-tune is neutral within its family; CORAL improves zero-shot in the strict panel only.

Per-class F1 for the CORAL model (Panel A): Meal_Preparation 0.88, Enter_Home 0.66, Eating 0.21, Leave_Home 0.17, Wash_Dishes 0.00. The negative transfer under pooling is distributed across classes rather than caused by a single dominant class; the rare Wash_Dishes activity (65 Aruba episodes) is not recovered by any zero-shot method.
